# Accounting for spatial trends in multi-environment diallel analysis in maize breeding

**DOI:** 10.1371/journal.pone.0258473

**Published:** 2021-10-21

**Authors:** Igor Ferreira Coelho, Marco Antônio Peixoto, Tiago de Souza Marçal, Arthur Bernardeli, Rodrigo Silva Alves, Rodrigo Oliveira de Lima, Edésio Fialho dos Reis, Leonardo Lopes Bhering

**Affiliations:** 1 Departamento de Biologia Geral, Universidade Federal de Viçosa (UFV), Viçosa, Minas Gerais, Brazil; 2 Departamento de Biologia, Universidade Federal de Lavras (UFLA), Lavras, Minas Gerais, Brazil; 3 Departamento de Agronomia, Universidade Federal de Viçosa (UFV), Viçosa, Minas Gerais, Brazil; 4 Instituto Nacional de Ciência e Tecnologia do Café (INCT Café), Universidade Federal de Lavras (UFLA), Lavras, Minas Gerais, Brazil; 5 Departamento de Agronomia, Universidade Federal de Jataí (UFJ), Jataí, Goiás, Brazil; KGUT: Graduate University of Advanced Technology, ISLAMIC REPUBLIC OF IRAN

## Abstract

Spatial trends represent an obstacle to genetic evaluation in maize breeding. Spatial analyses can correct spatial trends, which allow for an increase in selective accuracy. The objective of this study was to compare the spatial (SPA) and non-spatial (NSPA) models in diallel multi-environment trial analyses in maize breeding. The trials consisted of 78 inter-populational maize hybrids, tested in four environments (E1, E2, E3, and E4), with three replications, under a randomized complete block design. The SPA models accounted for autocorrelation among rows and columns by the inclusion of first-order autoregressive matrices (AR1 ⊗ AR1). Then, the rows and columns factors were included in the fixed and random parts of the model. Based on the Bayesian information criteria, the SPA models were used to analyze trials E3 and E4, while the NSPA model was used for analyzing trials E1 and E2. In the joint analysis, the compound symmetry structure for the genotypic effects presented the best fit. The likelihood ratio test showed that some effects changed regarding significance when the SPA and NSPA models were used. In addition, the heritability, selective accuracy, and selection gain were higher when the SPA models were used. This indicates the power of the SPA model in dealing with spatial trends. The SPA model exhibits higher reliability values and is recommended to be incorporated in the standard procedure of genetic evaluation in maize breeding. The analyses bring the parents 2, 10 and 12, as potential parents in this microregion.

## Introduction

Maize (*Zea mays* L.) is the most cultivated crop worldwide [1]. Quantitative traits, such as grain yield and plant height, are controlled by several genes and are highly influenced by the environment [2]. In this scenario, the genotype-by-environment (G × E) interaction, also known as phenotypic plasticity [3, 4], plays an essential role in phenotypic expression and can lead to difficulties in genetic selection [4].

Diallel mating designs are used for progeny tests [5] and are widely adopted in plant breeding [6–9]. These mating designs allow the evaluation of general and specific combining abilities, which are additive genetic effects based on general combining ability (GCA), and dominance genetic effect based on specific combining ability (SCA) [10, 11]. The GCA is given by the mean of the performance of a particular individual in combination with many others, and the SCA is the genetic effect of a specific cross [11, 12].

There are few examples of diallel multi-environment trials (MET) in maize breeding [13–15]. The mixed model methodology, or the residual maximum likelihood (REML)/best linear unbiased prediction (BLUP) [16, 17] procedure, has been widely adopted for analyzing MET in plant breeding [18–21]. However, diallel analyses are still underused [22], even being an effective procedure for genetic evaluation [23].

In diallel analyses, the REML/BLUP procedure allows the estimation of additive and dominance genetic variances, as well as the narrow- and broad-sense heritabilities [24–26]. In addition, it allows the prediction of additive and dominance genetic effects and the gains with selection, derived directly from parents and hybrid selection [22]. In MET analyses, the REML/BLUP procedure allows modeling different residuals [27–30] and genetic [18, 31–36] covariance structures and may be applied to unbalanced data [19, 37].

In diallel MET analyses, there are many sources of variation [38]. As main environmental sources, Burgueño et al. [39] cited soil structure, moisture, light interception, pathogen infections, and even crop management. To deal with spatial trends, one of the most adopted model structures is the separable first-order autoregressive (AR1⊗AR1), which considers a partitioned residual structure (*e*), composed of the correlated error (ξ) and, the independent random error (η) [40, 41], where *e* = *ξ*+*η*. For MET analyses, Smith et al. [31, 42] proposed the two-stage analysis, being stage 1 the modeling of AR1⊗AR1 spatial structure of residuals, to determine the best-fitted model. The output of stage 1 is imputed in stage 2, where the MET analysis is performed. This analysis has recently been applied in wheat [43] and soybean [44] breeding.

The two-stage analysis can present some issues, such as convergence failure and high computational demand, owing to the large amount of data [45]. Selective accuracy, heritability, and gain with selection can be used to compare different statistical models [46]. Selective accuracy indicates the precision of the predicted genotypic values [46], guides the strategies of breeding programs, and assists in decision-making [47].

There are several studies regarding diallel designs in maize breeding [14, 48–50], but only a few of them performed spatial analyses, mainly diallel MET analyses [51]. In addition, the use of spatial analyses has been increasing in recent years [43, 45, 52–57]. Therefore, the objective of this study was to compare the spatial (SPA) and non-spatial (NSPA) models for diallel MET analyses in maize breeding.

## Material and methods

### Genetic material

**Fig 1:**
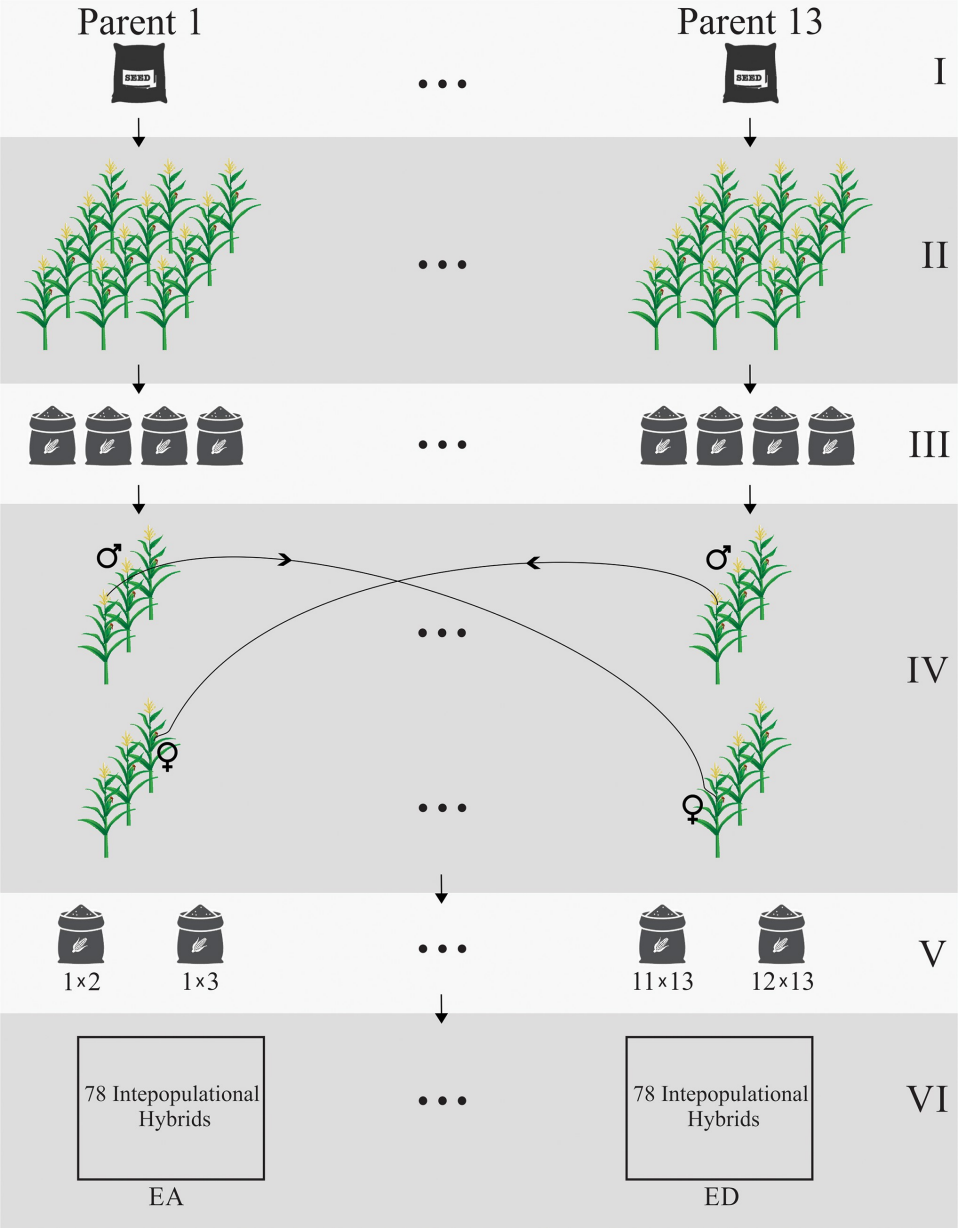
Interpopulational hybrids development process. The process occurred as following: (I) the most recommended commercial genetic materials (F_1_) were selected as parents 1 to 13; (II) these parents (F_1_) were cultivated in isolated fields; (III) the isolated fields were harvested and generated 12 F_2_ hybrids and one rustic genetic material; (IV) the F_2_ genetic materials were crossed artificially (controlled pollination) among themselves, in a diallel mating design; (V) as a result it was generated 78 interpopulational hybrids; and, (VI) the 78 interpopulational hybrids were cultivated in 4 environments (E1 to E4).

A diallel mating design (13 × 13), with no reciprocal crossing, was implemented (Fig 1 and S1 Table in S1 File). The genetic material consisted of 78 interpopulational hybrids evaluated in four environments (E1 to E4). Six commercial hybrids (F_1_), which were widely adopted in the region, were used as checks (S2 Table in S1 File).

### Experimental data

The trials were carried out during the 2018 winter season at four locations in the southwestern Goiás State, Brazil: the environments, E1 and E2, in the municipality of Jataí, and the environments, E3 and E4, in the municipalities of Caiapônia and Mineiros, respectively. The planting and harvesting dates, geographic coordinates, altitudes, average temperatures, and precipitation data for each environment are presented in S3 Table and S1 Fig in S1 File [58].

According to Alvares et al. [59], the weather in the southwestern region Goiás State is wet and temperate, with dry winters and hot summers (Cwa). The average annual temperature is around 21.5°C and the average rainfall is between 1.400 and 2.000 mm year^-1^. Agricultural practices for maize crops in Brazil [60] were adopted in this study and irrigation was not applied in the field (S3 Table in S1 File). The experiments were conducted in a randomized complete block design with three replications and 44 plants per plot. Plots were 4m long, with four rows spaced 0.45 m apart and a plot size of 7.2 m^2^.

To evaluate the grain yield (GY) trait, the ears at physiological maturity were harvested and shelled. Then, the grain weight and grain moisture percentage were recorded, and the GY trait was calculated at 13% moisture. All phenotypic measurements were taken from the middle rows, leaving the two border rows.

### Statistical analyses

The estimation of variance components and the prediction of genotypic values for the GY trait were made using the REML/BLUP procedure [16, 17], according to Gilmour et al. [61].

The individual analyses, considering the randomized complete block design with one observation per plot, was given by the following equation:

$$y=X\tau +Z_{g}u_{g}+Z_{s}u_{s}+e,$$ [1]

where **y** is the vector of phenotypic data, **τ** is the vector of replications and checks (assumed to be fixed) added to the general mean, **u**_*g*_ is the vector of the GCA effect (assumed as random), **u**_*s*_ is the vector of the SCA effect (assumed as random), and **e** is the vector of residuals (random). Uppercase letters refer to the incidence matrices for these effects.

In this model, $u_{g}N(0,\sigma_{g}^{2}),u_{s}N(0,\sigma_{s}^{2}),$ and *e N*(0,*R*); where $\sigma_{g}^{2}$ is the GCA variance (related to additive genetic variance), $\sigma_{s}^{2}$ is the SCA variance (related to dominance genetic variance), and *R* refers to the residual variance matrix. Models accounting for different residual variance structures considering the correlations among observations (rows and columns) were tested.

The joint analysis, considering the randomized complete block design with one observation per plot and four environments, was performed using the following equation:

$$y=X\tau +Z_{g}u_{g}+Z_{s}u_{s}+Z_{ge}u_{ge}+Z_{se}u_{se}+e,$$ [2]

where **y** is the vector of phenotypic data, **τ** is the vector of block-locations-checks combinations (assumed to be fixed), which comprises the effects of environment, replication within the environment, and checks added to the overall mean; **u**_*g*_ is the vector of additive genetic effects (assumed as random); **u**_*s*_ is the vector of dominant genetic effects (assumed as random); **u**_*ge*_ is the vector of the interaction between additive genetic effects and environments (random), **u**_*se*_ is the vector of the interaction between dominance genetic effects and environments (random), and *e* is the vector of residuals (random). Uppercase letters refer to the incidence matrices for these effects.

In this model: $u_{g}N(0,I\sigma_{g}^{2}),\;u_{s}N(0,I\sigma_{s}^{2}),\;u_{ge}N(0,{I\sigma }_{ge}^{2}),$ $u_{se}N(0,{I\sigma }_{se}^{2})$, and *eN*(0,*R*); where $\sigma_{g}^{2}$ is the GCA variance (related to additive genetic variance), $\sigma_{s}^{2}$ is the SCA variance (related to dominance genetic variance), $\sigma_{ge}^{2}$ is the GCA by environment interaction variance, $\sigma_{se}^{2}$ is the SCA by environment interaction variance, and *R* refers to the residual variance matrix. Models accounting for different residual variance structures considering the correlations among observations (rows and columns) were tested.

SPA and NSPA analyses were performed for comparison purposes.

#### Modelling non-genetic effects

In the individual analyses, the residual (co)variance structure could account for heterogeneity within the trial. The best fitted models were tested by the inclusion or the absence of the spatial information (given by rows and columns) in the fixed and random parts of the model. The joint analysis accounted for heterogeneity across environments in residual and genetic (co)variance matrices in two different ways: (i) by assuming spatially independent observations and (ii) by allowing spatial autocorrelations among observations, indexed by rows and columns.

The presence of autocorrelation among rows and columns was considered by the inclusion of first-order autoregressive matrices (**AR1**) for rows (*Σ*_*r*_(*ρ*_*r*_)) and columns (*Σ*_*c*_(*ρ*_*c*_)). There are three possible models beyond the NSPA model ($R=\sigma_{e}^{2}I_{r}⊗I_{c}$), where $\sigma_{e}^{2}$ is the residual variance, *I*_*r*_ is the identity matrix of rows (*r × r*), and *I*_*c*_ is the identity matrix of columns (*c × c*). The baseline model did not consider any correlations among the rows and columns. The following two residual structures account for the correlation among rows (M2) and columns (M3), respectively, by the inclusion of the AR1 structure. M2 and M3 contemplate the residual structure as $R=\sigma_{\xi }^{2}\Sigma_{r}(\rho_{r})⊗I_{c}$ and $R=\sigma_{\xi }^{2}I_{r}⊗\Sigma_{c}(\rho_{c})$, respectively. The last model accounts for correlations among rows and columns (M4), being $R=\sigma_{\xi }^{2}\Sigma_{r}(\rho_{r})⊗\Sigma_{c}(\rho_{c})$, where $\sigma_{\xi }^{2}$ is the SPA variance among columns and among rows, ⊗ is the Kronecker product, and, **AR1*x***, or, *Σ*_*x*_, represent first-order autoregressive correlation matrix, for ***x*** (row or column).

After selecting the best residual structure for each trial, the SPA information (row and column specifications) was included in the fixed and random parts of the model. First, the selected residual structure (MRx) of each trial was adopted as the first model, named Mx.1. Second, we tested the presence of fixed linear effects for rows and columns (Mx.2 and Mx.3). Third, we tested the presence of random linear effects for rows and columns (Mx.4 and Mx.5). Finally, the measurement error was added to the random part of the model (Mx.6).

The fixed part of the model (rows and columns) deals with rows and columns as covariates, and was developed as a regression of the phenotypic responses as a function of the row and column positions. In the random part of the model, the rows and columns effects were included to quantify the variability among rows and columns. Finally, we also tested the inclusion of an independent error regarding the measurement error, which is an independent residual variance, $\sigma_{\eta }^{2}$.

**Fig 2:**
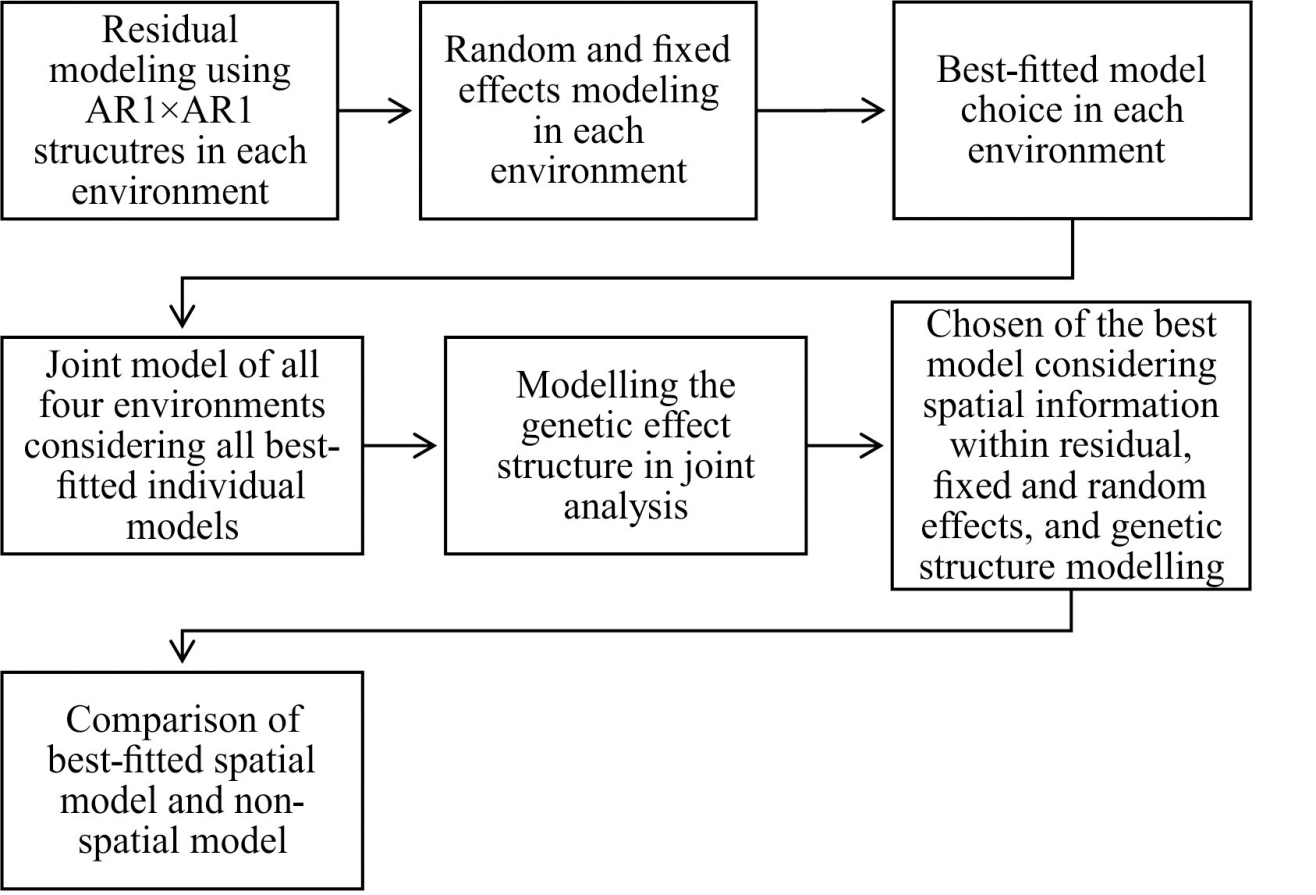
Flowchart of the spatial analysis in each trial and jointly. All steps of the spatial analysis by considering the spatial information (rows and columns) in the fixed and random parts of the model, in each trial. Following, it is considered all individual models to encounter the best joint model, and the most adequate genetic covariance structure in the multi-environment analysis.

Fig 2 presents the flowchart of all steps to encounter the best SPA models for each trial by modeling the fixed and random effects and to select the best genetic structure when analyzing all trials jointly in a MET analysis.

#### Modelling genetic effects

Five different genetic variance structures were tested across different environments: diagonal (DIAG), compound symmetry (CS), heterogeneous correlation (CORH), and factor analytic of first (FA1) and second (FA2) orders.

#### Model selection, significance test, and gain with selection

To select the best-fit model, the conditional Bayesian information criterion (BIC_c_) [62] was used. The comparison among models was based on the parameter vector (${\hat{\beta }}_{k}$), which stands for the covariance matrix to calculate the corrected *LogL* (*LogL*_*c*_), as follows:

$${BIC}_{c}=-2{LogL}_{c}+(p+q)ln\;(n),$$ [3]

where *LogL*_*c*_ is the corrected *LogL* by ${\hat{\beta }}_{k}{\hat{\beta }}_{k}$; *p* is the number of random parameters, *q* is the number of fixed parameters, and *n* is the model’s degrees of freedom.

The variances of the random effects were assessed using the likelihood ratio test (LRT) as follows [63]:

$$LRT=-2(logL_{R}-LogL),$$ [4]

where *LogL*_*R*_ is the logarithm of the maximum residual likelihood function of the reduced model. For the LRT test, chi-square statistics with 1 degree of freedom and 5% probability of Type I error were considered.

The additive genetic variance (${\hat{\sigma }}_{A}^{2}$), dominance genetic variance (${\hat{\sigma }}_{D}^{2}$), and broad-sense (H^2^) heritability (given by the additive genetic variance among hybrids) were estimated for each trial using the following equations [64–66]:

$${\hat{\sigma }}_{A}^{2}=4{\hat{\sigma }}_{g}^{2},$$ [5]

$${\hat{\sigma }}_{D}^{2}=4{\hat{\sigma }}_{s}^{2},\;\mathrm{and}$$ [6]

$$H^{2}=\frac{\frac{{\hat{\sigma }}_{A}^{2}}{2}+\frac{{\hat{\sigma }}_{D}^{2}}{4}}{\frac{{\hat{\sigma }}_{A}^{2}}{2}+\frac{{\hat{\sigma }}_{D}^{2}}{4}+{\hat{\sigma }}_{R}^{2}},$$ [7]

where ${\hat{\sigma }}_{g}^{2}$ and ${\hat{\sigma }}_{s}^{2}$ are the estimates of the general and specific combining ability variances, respectively.

In addition to the genetic parameters by each trial, the additive genetic variance (${\hat{\sigma }}_{A}^{2}$), dominance genetic variance (${\hat{\sigma }}_{D}^{2}$), additive by environment interaction variance (${\hat{\sigma }}_{AE}^{2}$), dominance by environment interaction variance (${\hat{\sigma }}_{DE}^{2}$), and broad-sense (H^2^) heritability, were estimated considering the four environments in a joint analysis using the following equations [64–66]:

$${\hat{\sigma }}_{A}^{2}=4{\hat{\sigma }}_{g}^{2},$$ [5]

$${\hat{\sigma }}_{D}^{2}=4{\hat{\sigma }}_{s}^{2},\;\mathrm{and}$$ [6]

$$H^{2}=\frac{\frac{{\hat{\sigma }}_{A}^{2}}{2}+\frac{{\hat{\sigma }}_{D}^{2}}{4}}{\frac{{\hat{\sigma }}_{A}^{2}}{2}+\frac{{\hat{\sigma }}_{D}^{2}}{4}+{\hat{\sigma }}_{AE}^{2}+{\hat{\sigma }}_{DE}^{2}+{\hat{\sigma }}_{R}^{2}},$$ [8]

In addition, the genotypic correlation across environments (*r*_*g*_) and the overall mean (*μ*_*g*_) for the GY trait was also calculated as follows:

$$r_{g}=\frac{{\hat{\sigma }}_{A}^{2}+{\hat{\sigma }}_{D}^{2}}{{\hat{\sigma }}_{A}^{2}+{\hat{\sigma }}_{AE}^{2}+{\hat{\sigma }}_{D}^{2}+{\hat{\sigma }}_{DE}^{2}}.$$ [9]

Selective accuracies ($r_{\hat{A}A}$ and $r_{\hat{D}D}$) were estimated for individual and joint ($r_{\hat{G}G}$) analyses using the following expression [67]:

$$r_{\hat{A}A}=\sqrt{1-\frac{PEV}{{\hat{\sigma }}_{A}^{2}}},$$ [10]

$$r_{\hat{D}D}=\sqrt{1-\frac{PEV}{{\hat{\sigma }}_{D}^{2}}},\;\mathrm{and},$$ [11]

$$r_{\hat{G}G}=\sqrt{1-\frac{\left[1-(1-\frac{PEV}{{\hat{\sigma }}_{A}^{2}})][1-(1-\frac{PEV}{{\hat{\sigma }}_{D}^{2}})\right]}{1-(1-\frac{PEV}{{\hat{\sigma }}_{A}^{2}})(1-\frac{PEV}{{\hat{\sigma }}_{D}^{2}})}}.$$ [12]

where *PEV* is the prediction error variance, according to the respective effect obtained by the diagonal elements of the inverse of the coefficient matrix of the mixed model equations.

The correlations among the NSPA and SPA models were evaluated using the Pearson correlation coefficient. Cohen’s Kappa coefficient (K) [68] was applied to estimate the agreement between the NSPA and SPA models. The agreement among the selected interpopulational hybrids was calculated as follows:

$$K=\frac{A-C}{D-C}x100,$$ [13]

where *A* is the number of matching selected interpopulational hybrids between the NSPA and SPA model rankings, *C* is the number of selected interpopulational hybrids due to chance (*C* = *bD*, where b is the selection intensity = 0.13), and *D* is the number of selected interpopulational hybrids (10).

#### Contrasting SPA and NSPA analyses

After selecting the individual and joint SPA models, further analyses were conducted using the NSPA model to expose the relative importance of the SPA model. Therefore, all analyses performed under the spatial approach were also re-made under a non-spatial approach, considering the independence among rows and columns.

#### Software

All statistical analyses were carried out using ASReml v.4.1 [61], ASReml-R [69], and R software [70].

## Results

### Modelling non-genetic effects

In trials E1 and E2, the NSPA model (MR1), which did not consider any correlation among the rows, and among the columns, was the best fitted model (Table 1). In trial E3, the best fitted model (MR4) considered the existence of correlation among rows and columns in the residual effects, while in trial E4, the best fitted model (MR3) considered the correlation among columns in the residual effects (Table 1).

**Table 1:** One-dimensional and two-dimensional autoregressive spatial models fitted for the grain yield trait.

Trials	Models	Variance Models	*p/q*	Full-LogL	BIC_c_
		Natural			
E1	MR.1	$\sigma_{R}^{2}I_{r}⊗I_{c}$	9/3	-1844.75	3755.21^#^
	MR.2	$\sigma_{\xi }^{2}\Sigma_{r}(\rho_{r})⊗I_{c}$	9/4	-1844.10	3759.39
	MR.3	$\sigma_{\xi }^{2}I_{r}⊗\Sigma_{c}(\rho_{c})$	9/4	-1841.98	3755.15
	MR.4	$\sigma_{\xi }^{2}\Sigma_{r}(\rho_{r})⊗\Sigma_{c}(\rho_{c})$	9/5	-1841.38	3759.42
E2	MR.1	$\sigma_{R}^{2}I_{r}⊗I_{c}$	9/3	-1845.73	3757.33^#^
	MR.2	$\sigma_{\xi }^{2}\Sigma_{r}(\rho_{r})⊗I_{c}$	9/4	-1844.05	3759.45
	MR.3	$\sigma_{\xi }^{2}I_{r}⊗\Sigma_{c}(\rho_{c})$	9/4	-1843.23	3757.82
	MR.4	$\sigma_{\xi }^{2}\Sigma_{r}(\rho_{r})⊗\Sigma_{c}(\rho_{c})$	9/5	-1841.51	3759.86
E3	MR.1	$\sigma_{R}^{2}I_{r}⊗I_{c}$	9/3	-1872.64	3811.04
	MR.2	$\sigma_{\xi }^{2}\Sigma_{r}(\rho_{r})⊗I_{c}$	9/4	-1863.07	3797.39
	MR.3	$\sigma_{\xi }^{2}I_{r}⊗\Sigma_{c}(\rho_{c})$	9/4	-1852.78	3776.81
	MR.4	$\sigma_{\xi }^{2}\Sigma_{r}(\rho_{r})⊗\Sigma_{c}(\rho_{c})$	9/5	-1846.40	3769.53^#^
E4	MR.1	$\sigma_{R}^{2}I_{r}⊗I_{c}$	9/3	-1807.35	3680.42
	MR.2	$\sigma_{\xi }^{2}\Sigma_{r}(\rho_{r})⊗I_{c}$	9/4	-1801.14	3673.46
	MR.3	$\sigma_{\xi }^{2}I_{r}⊗\Sigma_{c}(\rho_{c})$	9/4	-1797.21	3665.61^#^
	MR.4	$\sigma_{\xi }^{2}\Sigma_{r}(\rho_{r})⊗\Sigma_{c}(\rho_{c})$	9/5	-1793.83	3664.33

The variance model includes just the Natural, which means the residual effects. Number of fixed parameters (*p*), number of variance parameters (*q*), the full log-likelihoods (Full-LogL), and Conditional Bayesian information criteria (BIC_c_), are given for the spatial models fitted for each trial.

^#^: best-fitted model; $\sigma_{R}^{2}$ and $\sigma_{\xi }^{2}$: variance components of units and correlated residuals; *I*_*r*_ and *I*_*c*_: identity matrices of rows and columns; *Σ*_*r*_(*ρ*_*r*_) and *Σ*_*c*_(*ρ*_*c*_): correlation matrices for the row model (order *r*, autocorrelation parameter *ρ*_*r*_) and column model (order *c*, autocorrelation parameter *ρ*_*c*_).

### Modelling fixed and random effects

The E1, E2, and E3 trials did not consider any other effect beyond the selected residual in the previous step; the E4 trial was the only trial that considered the inclusion of a linear effect of rows (M3.2), which improved the model’s goodness-of-fit (Table 2).

**Table 2:** Spatial models fitted for the grain yield trait.

Trials	Models	Variance Models		(*p*/*q*)	Full-LogL	BIC_c_
		Global/Extraneous	Natural			
E1	M1.1		$\sigma_{R}^{2}I_{r}⊗I_{c}$	9/3	-1841.98	3755.15^#^
	M1.2	*β*_*r*_×**R**	$\sigma_{R}^{2}I_{r}⊗I_{c}$	10/3	-1841.84	3760.29
	M1.3	*β*_*r*_×**R**, *β*_*c*_×**C**	$\sigma_{R}^{2}I_{r}⊗I_{c}$	11/3	-1841.99	3766.12
	M1.4	*β*_*r*_×**R**, *β*_*c*_×**C**, $\sigma_{r}^{2}I_{r}$	$\sigma_{R}^{2}I_{r}⊗I_{c}$	11/4	-1841.99	3771.59
	M1.5	*β*_*r*_×**R**, *β*_*c*_×**C**, $\sigma_{r}^{2}I_{r},\sigma_{r}^{2}I_{c}$	$\sigma_{R}^{2}I_{r}⊗I_{c}$	11/5	-1841.99	3777.07
	M1.6	*β*_*r*_×**R**, *β*_*c*_×**C**, $\sigma_{r}^{2}I_{r},\sigma_{r}^{2}I_{c}$	$\sigma_{R}^{2}I_{r}⊗I_{c},\sigma_{R}^{2}I_{n}$	11/6	-1844.75	3782.59
E2	M1.1		$\sigma_{R}^{2}I_{r}⊗I_{c}$	9/3	-1845.73	3757.33^#^
	M1.2	*β*_*r*_×**R**	$\sigma_{R}^{2}I_{r}⊗I_{c}$	10/3	-1845.63	3762.56
	M1.3	*β*_*r*_×**R**, *β*_*c*_×**C**	$\sigma_{R}^{2}I_{r}⊗I_{c}$	11/3	-1845.74	3768.32
	M1.4	*β*_*r*_×**R**, *β*_*c*_×**C**, $\sigma_{r}^{2}I_{r}$	$\sigma_{R}^{2}I_{r}⊗I_{c}$	11/4	-1844.19	3770.71
	M1.5	*β*_*r*_×**R**, *β*_*c*_×**C**, $\sigma_{r}^{2}I_{r},\sigma_{r}^{2}I_{c}$	$\sigma_{R}^{2}I_{r}⊗I_{c}$	11/5	-1840.20	3768.22
	M1.6	*β*_*r*_×**R**, *β*_*c*_×**C**, $\sigma_{r}^{2}I_{r},\sigma_{r}^{2}I_{c}$	$\sigma_{R}^{2}I_{r}⊗I_{c},\sigma_{R}^{2}I_{n}$	11/6	-1840.20	3773.71
E3	M4.1		$\sigma_{\xi }^{2}\Sigma_{r}(\rho_{r})⊗\Sigma_{c}(\rho_{c})$	9/5	-1846.40	3769.53^#^
	M4.2	*β*_*r*_×**R**	$\sigma_{\xi }^{2}\Sigma_{r}(\rho_{r})⊗\Sigma_{c}(\rho_{c})$	10/5	-1845.31	3772.76
	M4.3	*β*_*r*_×**R**, *β*_*c*_×**C**	$\sigma_{\xi }^{2}\Sigma_{r}(\rho_{r})⊗\Sigma_{c}(\rho_{c})$	11/5	-1846.55	3780.79
	M4.4	*β*_*r*_×**R**, *β*_*c*_×**C**, $\sigma_{r}^{2}I_{r}$	$\sigma_{\xi }^{2}\Sigma_{r}(\rho_{r})⊗\Sigma_{c}(\rho_{c})$	11/6	-1841.86	3776.88
	M4.5	*β*_*r*_×**R**, *β*_*c*_×**C**, $\sigma_{r}^{2}I_{r},\;\sigma_{r}^{2}I_{c}$	$\sigma_{\xi }^{2}\Sigma_{r}(\rho_{r})⊗\Sigma_{c}(\rho_{c})$	11/7	-1841.86	3782.36
	M4.6	*β*_*r*_×**R**, *β*_*c*_×**C**, $\sigma_{r}^{2}I_{r},\sigma_{r}^{2}I_{c}$	$\sigma_{\xi }^{2}\Sigma_{r}(\rho_{r})⊗\Sigma_{c}(\rho_{c}),\;\sigma_{R}^{2}I_{n}$	11/8	-1841.87	3787.86
E4	M3.1		$\sigma_{\xi }^{2}I_{r}⊗\Sigma_{c}(\rho_{c})$	9/5	-1793.83	3664.33
	M3.2	*β*_*r*_×**R**	$\sigma_{\xi }^{2}I_{r}⊗\Sigma_{c}(\rho_{c})$	10/5	-1783.50	3649.07^#^
	M3.3	*β*_*r*_×**R**, *β*_*c*_×**C**	$\sigma_{\xi }^{2}I_{r}⊗\Sigma_{c}(\rho_{c})$	11/5	-1786.03	3659.67
	M3.4	*β*_*r*_×**R**, *β*_*c*_×**C**, $\sigma_{r}^{2}I_{r}$	$\sigma_{\xi }^{2}I_{r}⊗\Sigma_{c}(\rho_{c})$	11/6	-1785.88	3664.86
	M3.5	*β*_*r*_×**R**, *β*_*c*_×**C**, $\sigma_{r}^{2}I_{r},\sigma_{r}^{2}I_{c}$ *β*_*r*_×**R**, *β*_*c*_×**C**, $\sigma_{r}^{2}I_{r},\;\sigma_{r}^{2}I_{c}$	$\sigma_{\xi }^{2}I_{r}⊗\Sigma_{c}(\rho_{c})$	11/7	-1785.88	3670.34
	M3.6		$\sigma_{\xi }^{2}I_{r}⊗\Sigma_{c}(\rho_{c}),\sigma_{R}^{2}I_{n}$	11/8	-1783.70	3671.44

Number of fixed parameters (*p*), number of variance parameters (*q*), the full log-likelihoods (Full-LogL) and the conditional Bayesian Information Criteria (BIC_c_), are given for the spatial models fitted for each trial.

^#^: best-fitted model; $\sigma_{r}^{2},\;\sigma_{c}^{2},\;\sigma_{R}^{2}$, and $\sigma_{\xi }^{2}$: variance components of rows, columns, units, and correlated residuals, respectively; *I*_*r*_, *I*_*c*_, and *I*_*n*_: identity matrices of rows, columns, and units, respectively; *β*_*r*_ and *β*_*c*_: liner regression coefficient for rows (**R**) and columns (**C**), respectively; *Σ*_*r*_(*ρ*_*r*_) and *Σ*_*c*_(*ρ*_*c*_): correlation matrices for the row model (order *r*, autocorrelation parameter *ρ*_*r*_) and column model (order *c*, autocorrelation parameter *ρ*_c_).

### Modelling the genetic effects in multi-environment trials

After selecting the best-fitted model for each trial, a joint analysis was performed, considering the best-fitted models, indicated by *BIC*_*c*_ in the previous steps. In the joint analysis, the genetic (additive and dominance effects) covariance matrix was also modeled. The compound symmetry model (CS) was assigned as the best-fit model (Table 3).

**Table 3:** Models fitted for the multi-environment trial for the grain yield trait.

Models^‡^	p	q	Full-LogL	BIC_c_
DIAG	37	15	-7328.15	15013.38
CS	37	11	-7309.11	14947.82^#^
CORH	37	17	-7305.91	14982.64
FA1	37	23	-7306.00	15024.01
FA2	37	29	-7297.82	15048.86

Number of fixed parameters (*p*), number of variance parameters (*q*), the full log-likelihoods (Full-LogL) and the conditional Bayesian information criterion (BIC_c_).

^#^: best-fitted model

^‡^Models: DIAG: diagonal, CS: compound symmetry, CORH: heterogeneous correlation, FA: Factor Analytic of first order (FA1), and Factor Analytic of second order (FA2).

### Estimates of variance components, heritability, and coefficient of determination of the dominance effects

**Fig 3:**
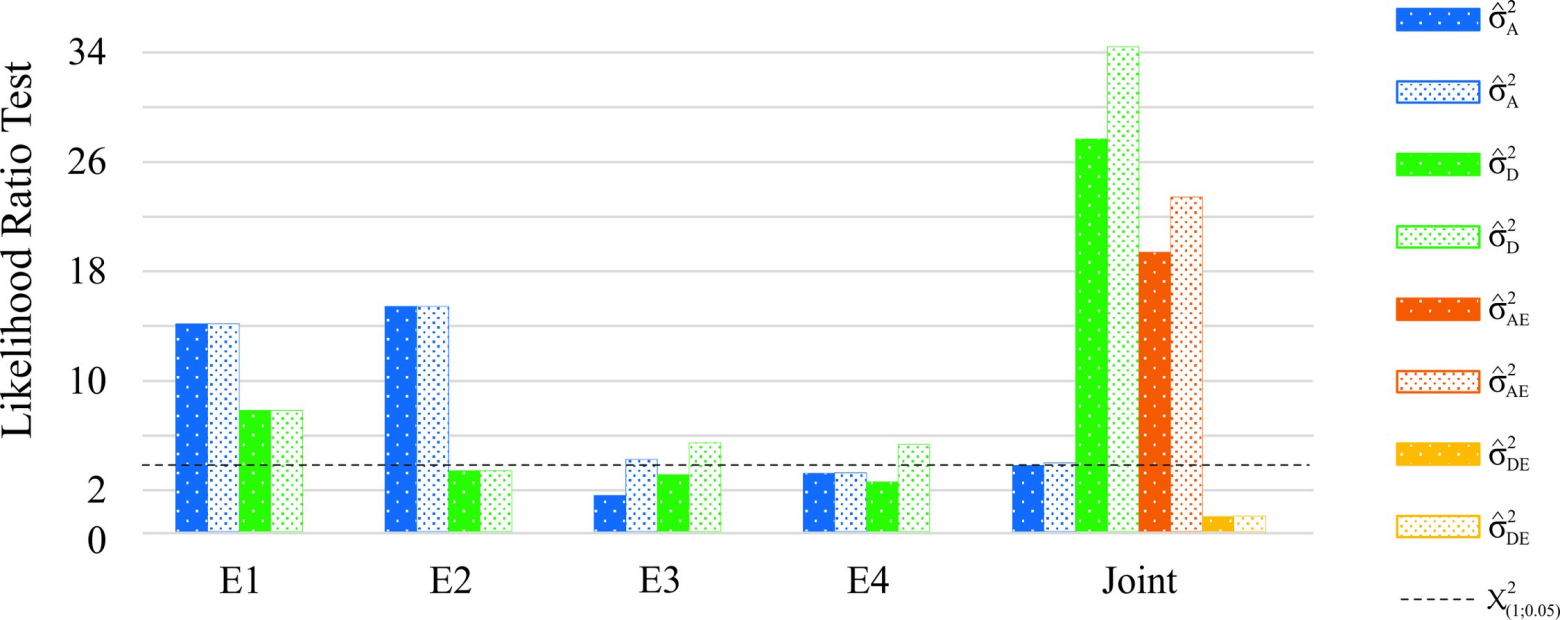
Likelihood ratio test for spatial and non-spatial analyses. Likelihood ratio test (LRT), using the Chi-square test with 1 degree of freedom (p< 0.05) (represented by the dashed line), for the genetic (additive and dominance) effects and their interaction with environment (in joint analysis) for (a) non-spatial and (b) spatial analyses.

The LRT results for both the SPA and NSPA analyses are presented in Fig 3. We observed differences in the significance of the random effects, where the SPA and NSPA models were considered, beyond the additive by environment interaction and dominance genetic effects, the additive genetic effects (GCA) were significant. When analyzing each trial (Fig 3), the same pattern was observed for trials E3 and E4, where, after SPA, genetic effects were considered significant and were not considered by NSPA.

The SPA and NSPA models used for the joint analyses fit the compound symmetry structure (Table 4). The NSPA model fitted a homogeneous residual structure, while SPA considered all required spatial information for each trial in the residual structure, as well as fixed and random effects.

**Table 4:** Variance components, genetic parameters and phenotypic mean for the four trials (individual and joint analyses), by non-spatial (NSPA) and spatial (SPA) analyses.

Variance Components / Genetic Parameters	E1		E2		E3		E4		Joint	
	NSPA	SPA	NSPA	SPA	NSPA	SPA	NSPA	SPA	NSPA	SPA
${\hat{\sigma }}_{A}^{2}$	148290.5*	148290.5*	117052.3*	117052.3*	34659.3	49338.4*	33305.9	32158.0	36901.36	36459.54*
${\hat{\sigma }}_{D}^{2}$	215948.2*	215948.2*	114923.1	114923.1	161769.3	158303.6*	94081.3	103489.8*	126732.59*	130537.40*
${\hat{\sigma }}_{AE}^{2}$	-		-		-		-		45903.86*	47679.46*
${\hat{\sigma }}_{DE}^{2}$									21115.96	9071.981
${\hat{\sigma }}_{R}^{2}$	856498.4	856498.4	760842.2	760842.2	1129629.3	1098305.3	705513.7	590915.9	862757.39	-
${\hat{\sigma }}_{R_{1}}^{2}$	-		-		-		-		-	934131.2
${\hat{\sigma }}_{R_{2}}^{2}$										740443.1
${\hat{\sigma }}_{R_{3}}^{2}$										1107782.70
${\hat{\sigma }}_{R_{4}}^{2}$										572230.99
${\hat{H}}^{2}$	0.1301	0.1301	0.1029	0.1029	0.0487	0.0553	0.0539	0.0663	0.0512	-
${\hat{H}}_{1}^{2}$	-		-		-		-		-	0.0488
${\hat{H}}_{2}^{2}$										0.0600
${\hat{H}}_{3}^{2}$										0.0418
${\hat{H}}_{4}^{2}$										0.0748
$r_{\hat{G}G}$	0.8648	0.8648	0.8628	0. 8628	0.7059	0.7903	0.7489	0.7745	0.8334	0.8453
$r_{\hat{A}A}$	0.8391	0.8391	0.8472	0.8472	0.6168	0.7274	0.6949	0.7117	0.7130	0.7183
$r_{\hat{D}D}$	0.6085	0.6085	0.5201	0.5201	0.5242	0.5922	0.5055	0.5663	0.7362	0.7705
*r*_*G*_									0.7094	0.7464
*μ*_*g*_	5452.49		5871.85		5697.91		3849.46		5221.57	

E1: trial 1, E2: trial 2, E3: trial 3, E4: trial 4, and Joint: Joint analysis.

*: Significantly at 0.05 Type I probability of error by the chi-square test; ${\hat{\sigma }}_{A}^{2}$: additive genetic variance; ${\hat{\sigma }}_{D}^{2}$: dominance genetic variance; ${\hat{\sigma }}_{AE}^{2}$: additive by environment interaction variance; ${\hat{\sigma }}_{AE}^{2}$: dominance by environment interaction variance; ${\hat{\sigma }}_{R}^{2}$: residual variance; ${\hat{H}}^{2}$: broad-sense heritability; $r_{\hat{G}G}$: selective accuracy for genotypic effect; $r_{\hat{A}A}$: selective accuracy for genetic additive effect; $r_{\hat{D}D}$: selective accuracy for dominance genetic effect; *r*_*G*_: genotypic correlation across environments; *μ*_*g*_: overall mean.

Differences between SPA and NSPA analyses were observed for the estimates of additive and dominance genetic variances and residual variance in each trial (Table 4). In the joint analyses, where the additive by environment interaction (${\hat{\sigma }}_{AE}^{2}$) and dominance by environment interaction (${\hat{\sigma }}_{DE}^{2}$) variances were estimated, the NSPA and SPA also conducted different estimates (Table 4). Trials E1, E3, and E4 presented higher dominance genetic variances than additive genetic variances (Table 4). Conversely, trial E2 presented a higher additive genetic variance (Table 4). The joint analyses conducted to similar genetic variance estimates among NSPA and SPA, except for dominance by environment interaction variances that were 50% lower in NSPA (Table 4). The residual structure, which was homogeneous in NSPA, became heterogeneous in SPA, and it was possible to obtain residual variances per environment. Regarding the residual variance, E3 and E4 trials presented reduced values in SPA, achieving a reduction of 20% in the E4 trial when adopting the indicated SPA models over the NSPA model (Table 4).

In individual analyses, heritability estimates varied across trials. Considering the broad-sense heritabilities, trials E1 and E2 presented estimates superior to trials E3 and E4. In trial E3, SPA showed an increase of approximately 1.2% over the NSPA estimate. Broad-sense heritability presented estimates that were superior to 15% in all trials. In addition, trials E1 and E2 had the same estimates in the SPA and NSPA analyses, since they did not require any spatial information. In trials E3 and E4, the broad-sense heritability increased by 1.1% and 3%, respectively, due to the adoption of SPA instead of NSPA models.

The selective accuracies ($r_{\hat{G}G},\;r_{\hat{A}A}$, and, $r_{\hat{D}D}$) varied from NSPA to SPA analyses in each trial. E1 and E2 presented the same values in the NSPA and SPA analyses, which were superior to 0.80 the selective accuracy of genotypic effects and selective accuracy for genetic additive effects. The selective accuracies for dominance genetic effect were 0.60 and 0.52 in the trials E1 and E2, respectively. The trials that required spatial information (E3 and E4) had increased accuracy values from the NSPA to SPA analyses. The highest difference was observed in the trial E3, where the selective accuracy for genetic additive effects increased from 0.62 to 0.73 when considered the spatial information. The same behaviors were observed in the accuracy estimates in trials E3 and E4.

The joint analyses showed improvement of the genetic parameter estimates (heritability, accuracy, and genotypic correlation) from NSPA to SPA analyses. First, the NSPA analyses presented broad-sense heritability estimates of approximately 5%. For SPA analysis, the broad-sense heritability estimates were split per environment, and their values varied according to each environment, from 4% (E1 and E3) to over 7% (E4). Compared to the individual analysis, the broad-sense heritability decreased from 13% in trial E1 to 4.8% in joint analysis, but in trial E4, it increased from 6.6% to 7.5%. The genotypic correlation across environments increased from 0.71, in NSPA analysis, to 0.75, in SPA analysis.

### Contrasting additive and dominance genetic effects rankings

**Fig 4:**
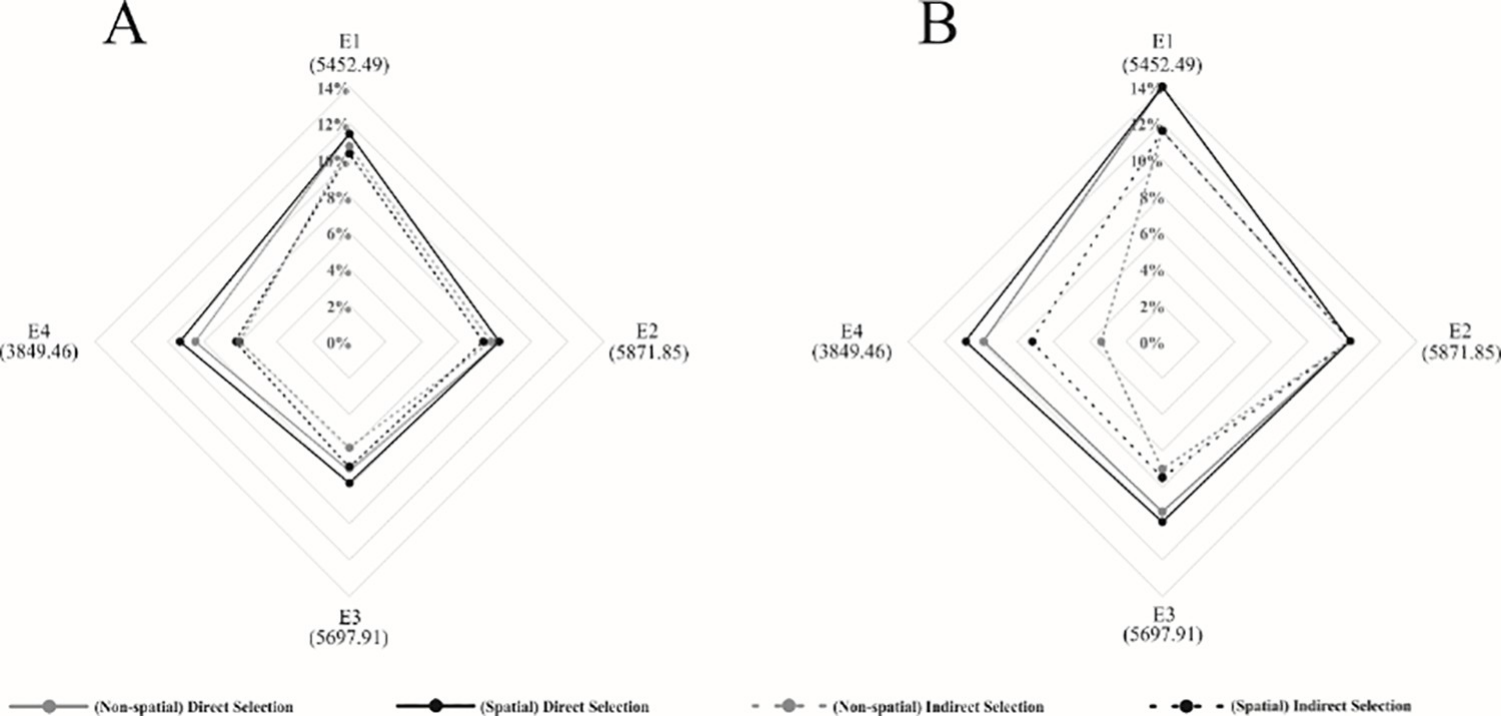
Selection gains for 10 (A) and 5 (B) selected hydrids. Direct gain, considering spatial and non-spatial analyses, and indirect gain, given by joint analysis, in both approaches, in all four environments. The values between parentheses indicated the predicted mean of each environment (E1 = trial 1, E2 = trial 2, E3 = trial 3, E4 = trial 4).

We contrasted the additive and dominance genetic effects by NSPA and SPA analyses, in each trial, to check the differences when the spatial information was used. Considering the three best parents selected, the same selected parents were observed in trials E1 and E2 (P1, P10, and P12) (S4 Table in S1 File). In trial E3, only one parent (P6) was identified among the top three highest additive genetic values in both NSPA and SPA. In trial E4, two parents were the same, P6 and P12, in the NSPA and SPA analyses. Based on the predicted additive and dominance genetic effects, it was also possible to predict the genotypic effects of the interpopulational hybrids in each trial (S5 Table in S1 File) and the gains with selection (Fig 4) by NSPA and SPA analyses.

The predicted dominance genetic effects of the parents’ combinations (S6 Table in S1 File), represented by the interpopulational hybrids (H*i*,*j*, where *i* and *j* the parents crossed). The top ten genetic materials were compared and detached. The E3 and E4 trials presented different crossings into the top 10 dominance genetic values. The Pearson correlation coefficients were equal to 1.0 in the E1 and E2 trials, while in the E3 and E4 trials, they were higher than 0.8 (S6 Table in S1 File). The Cohen’s Kappa coefficients presented the same results in the E1 and E2 trials (1.0), while in E3 and E4 trials they were around 0.60 (S6 Table in S1 File).

## Discussion

The use of statistical methods in breeding programs enables the breeder to access all information available from simpler, as phenotypic data, to complex inputs, such as genomic relationships and spatial correlations [71–73]. The accuracy tends to increase by modeling more sources of information, leading to more efficient use of resources [74].

According to Cooper et al. [75], there are some variations among plots that the breeder cannot predict *a priori*, even when the soil fertility is well controlled and the experimental design is appropriate. These spatial trends contribute to a large number of non-target signals [76]. Indifference or proper accounting for exogenous sources of variation decreases the accuracy [73, 77] and selection mistakes [41, 78].

The occurrence of diseases and insects within and across blocks, missing plots, irrigation misdistribution, and micro soil fertility spots can affect the residual independence among plots [79]. In this manner, trials E3 and E4, which required first-order autoregressive (AR1) modeling for rows and/or columns, also presented the lowest values for broad-sense heritability, selective accuracy, and phenotypic means for the GY trait.

So and Edwards [29] found, in a simulated balanced dataset study, similar results compared compound symmetry and unstructured models. This statement is relevant because of the genotypic correlation estimate across environments (0.75), by SPA analysis, where the compound symmetry structure, even being a more conservative structure [36], is capable of dealing with the level of G×E interaction effect without loss of predictive capability.

When comparing the NSPA and SPA models, the results demonstrated the importance of spatial analysis. Relevant studies on potato [80] and soybean [44] also presented satisfactory results when spatial trends were considered. Higher selective accuracies were encountered in the SPA models, reinforcing the importance and potential of the SPA analyses in crop breeding. Another simulation study of soybean breeding considering the accounting of spatial information in the model also presented gains in selective accuracy when autoregressive structures were used for spatial information [76].

The significance of the random effects tested by the LRT differed when spatial information was considered in both individual and joint analyses. The selective accuracies and genetic gains demonstrate the capacity of data correction, when necessary, by spatial analyses. Similar results were reported by Resende [11], who affirmed that the presence of spatial dependence is required when there is any kind of heterogeneity in the experiment, or even inside the block. Furthermore, by the adoption of SPA analyses, when applicable, it was possible to estimate higher values of genetic variances and parameters, which proves the importance of considering the best-fitted model. For instance, in trial E3, after modeling the residuals and including spatial information in the model, it was possible to obtain a higher portion of the additive and dominance genetic effects, turning them into significant effects. They were non-significant in the NSPA analyses and became significant in the SPA analyses. In addition, the genetic variances, heritabilities, and selective accuracies were maximized using SPA analyses. The genotype ranking also varied between the NSPA and SPA analyses. This information can be used in breeding programs for genetic selection purposes.

Selective accuracy demonstrates the correlation between the predicted and true genetic values. As mentioned previously, there was an increase from NSPA to SPA analyses. These results reinforce the superiority of SPA analyses, which maximize the selective accuracy when compared to NSPA analyses. Residual modeling considering the dependence among rows and/or columns decreased the residual variance within the trials. The consideration of two errors, dependent and independent, allows modeling of the dependence part and mitigates the residue in explained portions. This error investigation maximized the selective accuracies in the environments, which indicated the spatial models as the best-fitted model because of the possibility of modeling more environmental effects that were not considered in the NSPA analyses.

One of the main goals of plant breeding programs is to select the best genotypes to increase genetic gains [81]. In addition, to maximize the frequency of favorable alleles for a specific trait, plant breeders use the best-ranked genotypes in the selection process to develop hybrids [81]. Following this idea, the selection gains based on the predicted genotypic effects by the BLUP method confirm the corrective capacity of SPA analyses over the NSPA analyses. The predicted genetic values can be used to predict the breeding value of individuals [82]. The joint analysis allows the calculation of the additive genetic effect and its interaction with the environment when considering all four environments. This fact increases the accuracy of the selection gain, as it is based on the additive genetic effect free of the GxE interaction effect. Herein, we confirmed the corrective capacity of spatial analyses when considering the selection gains, as well the genotype ranking, which led to the selection of the right genotypes in the SPA analyses ranking.

Furthermore, the differences between SPA and NSPA analyses rankings, in trials E3 and E4 (which spatial information was used) reinforce the importance of this analysis. It was observed that the spatial information was useful for selecting different parents and interpopulational hybrids using SPA over NSPA analyses. In addition, using the Pearson correlation coefficient demonstrates the existence of different genetic materials when contrasting NSPA and SPA analyses, and the Cohen’s Kappa coefficient, which focuses on the selected genetic materials, confirms the difference among the selected.

By observing the differences between the additive and dominance genetic effects, some interesting conclusions can be drawn for use in maize breeding programs. For instance, in trial E1, the H10,12, from the best parents (by additive effects) also presented a high dominance genetic effect; however, in trial E2, this effect was negative, demonstrating the presence of dominance by environment interaction effect, and its importance to calculate it. P6, which showed one of the lowest additive genetic effects, when combined with P12 (one of the highest additive genetic effects) presented one of the highest dominance genetic effects. These characteristics were observed in all the trials. In trial E3, a significant difference was noted between variance estimates and genetic parameter values from NSPA and SPA analyses, which demonstrates the importance of the SPA analyses in this study. Focusing on P3 and P12 as an example, the P3 was into three better parents in NSPA analyses, and decreased significantly in SPA analyses, while P12 arose from a median rank (in NSPA) to the third better in SPA analyses. Following, when looking into the dominance genetic effects just of the same two parents cross (H3,12), it was in the top 10 in the NSPA analyses, and dropped to the 16^th^ position in SPA analyses. As demonstrated, the spatial trend corrections affected the additive and dominance genetic effects and must be considered by the breeder in breeding program strategies. The selection will be made based on these genotypic, additive genetic, and dominance genetic effects values, and their accuracy will directly affect the subsequent cycles of the breeding process.

Identifying the best genetic material is the main goal of any breeding program. The main goal of this study was to identify the best potential parents and their combinations to form heterotic groups. The interpretation individually and jointly brings the parents 2, 10 and 12, as potential parents in this microregion composted by the four environments. Overall, they presented a high additive genetic effect (meaning they have a high proportion of favorable alleles to GY trait) and a high dominance genetic effect (seem to be contrasting and complementary), which would be very interesting in a hybrid composition, as the heterosis effect is expected in maize.

In this way, this study highlighted the importance of considering the dependence among rows and columns when required for specific trials and applied all additional information into the joint analysis, combined with the best genetic covariance structure. The adoption of SPA analyses led to better results, as observed in BICc, selective accuracy, heritability, and selection gains. This study confirmed the importance of SPA diallel MET analyses in maize breeding, which is a pioneer in the literature.

## Conclusion

The adoption of MET permits a better understanding of the genetic effect, as it enables the breeder to account for the GxE interaction effect. This makes it possible to isolate the genetic effect or further explore the GxE interaction effect, depending on the goal. In addition, the diallel matching design enriches the study as it accesses the effects within the genotypic effect, both additive and dominance genetic effects, by crossing all parents among themselves. We found that the SPA analyses maximize the selective accuracy when compared to NSPA analyses, confirming the superiority of the SPA models. In addition, the LRT confirmed that the mismodeling process can lead to the misinterpretation of model effects, where some significant effects can be considered non-significant due to inappropriate analyses.

Based on the fitted information, it was possible to identify the genotypes with the highest potential for future heterotic groups that can be used as potential commercial hybrids. The parents 2, 10 and 12, were indicated as potential parents in this microregion. Owing to their high additive genetic effects, bringing favorable genes to the future parental lines, their positive combination, given by the dominance genetic effect, will be expressed as a strong heterosis in future hybrids.

## Supporting information

S1 File Tables and figure to aid the understanding of the manuscript’s sections (materials and methods, and results). (DOCX)

S1 Dataset File containing the dataset. (TXT)

## References

[1] USDA. Economic Research Service. 2019.

[2] Allard RW. Principles of plant breeding. São Paulo—SP: Edgard Blücher; 1971.

[3] Resende MDV. Genética quantitativa e de populações. 1st ed. Suprema, Visconde do Rio Branco. Visconde do Rio Branco: Suprema; 2015.

[4] Marchal A, Schlichting CD, Gobin R, Balandier P, Millier F, Muñoz F, et al. Genotype by environment interactions in forest tree breeding: review of methodology and perspectives on research and application. Plant Genome. 2019;13: 281–288. doi: 10.1017/S0021859605005587

[5] Dong L, Xie Y, Sun X. Full-diallel-based analysis of genetic parameters for growth traits in Japanese larch (Larix kaempferi). New For. 2020;51: 261–271. doi: 10.1007/s11056-019-09729-6

[6] Melani MD, Carena MJ. Alternative Maize Heterotic Patterns for the Northern Corn Belt. Crop Sci. 2005;45: 2186–2194. doi: 10.2135/cropsci2004.0289

[7] Rodrigues MC, Chaves LJ, Pacheco CAP. Heterosis in crosses among white grain maize populations with high quality protein. Pesqui Agropecu Bras. 2006;41: 59–66. doi: 10.1016/j.jchromb.2006.03.061 16697280

[8] Welcker C, Thé C, Andréau B, De Leon C, Parentoni SN, Bernal J, et al. Heterosis and Combining Ability for Maize Adaptation to Tropical Acid Soils—Implications for Future Breeding Strategies. Crop Sci Madison. 2005;45: 2405–2413.

[9] Coelho IF, Alves RS, Rocha JR do AS de C, Peixoto MA, Teodoro LPR, Teodoro PE, et al. Multi-trait multi-environment diallel analyses for maize breeding. Euphytica. 2020;216: 144. doi: 10.1007/s10681-020-02677-9

[10] Zhang X, Lv L, Lv C, Guo B, Xu R. Combining Ability of Different Agronomic Traits and Yield Components in Hybrid Barley. Zhou M, editor. PLoS One. 2015;10: 9. doi: 10.1371/journal.pone.0126828 26061000 PMC4465181

[11] Resende MDV de. Genética biométrica e estatística no melhoramento de plantas perenes. Colombo: Embrapa Florestas; 2002.

[12] Sprague GF, Tatum LA. General vs. Specific Combining Ability in Single Crosses of Corn 1. Agron J. 1942;34: 923–932. doi: 10.2134/agronj1942.00021962003400100008x

[13] Wegary D, Vivek BS, Labuschagne MT. Combining ability of certain agronomic traits in quality protein maize under stress and nonstress environments in Eastern and Southern Africa. Crop Sci. 2014;54: 1004–1014. doi: 10.2135/cropsci2013.09.0585

[14] Nardino M, Souza VQ De, Baretta D, Konflanz VA, Follmann DN, Carvalho IR, et al. Partial diallel analysis among maize lines for characteristics related to the tassel and the productivity. African J Agric Res. 2016;11: 15–20. doi: 10.5897/AJAR2014.10314

[15] Faria MV, Mendes MC, Rossi ES, Junior OP, Rizzardi DA, Gralak E, et al. Análise dialélica da produtividade e do progresso da severidade de doenças foliares em híbridos de milho em duas densidades populacionais. Semin Ciência Agrárias. 2015;36: 123–134. doi: 10.5433/1679-0359.2015v36n1p123

[16] Henderson CR. Best linear unbiased estimation and prediction under a selection model. Biometrics. 1975;31: 423–447. Available: https://www.jstor.org/stable/pdf/2529430.pdf 1174616

[17] Patterson HD, Thompson R. Recovery of inter-block information when block sizes are unequal. Biometrika. 197158: 545–554. doi: 10.1093/biomet/58.3.545

[18] Burgueño J, Crossa J, Cotes JM, Vicente FS, Das B. Prediction Assessment of Linear Mixed Models for Multienvironment Trials. Crop Sci. 2011;51: 944–954. doi: 10.2135/cropsci2010.07.0403

[19] Smith AB, Cullis BR, Thompson R. The analysis of crop cultivar breeding and evaluation trials: An overview of current mixed model approaches. J Agric Sci. 2005;143: 449–462. doi: 10.1017/S0021859605005587

[20] Piepho H, Möhring J. Selection in Cultivar Trials—Is It Ignorable? Crop Sci. 2006;46: 192–201. doi: 10.2135/cropsci2005.04–0038

[21] Peixoto MA, Coelho IF, Evangelista JSPC, Alves RS, do Amaral Santos de Carvalho Rocha JR, Farias FJC, et al. Reaction norms‐based approach applied to optimizing recommendations of cotton genotypes. Agron J. 2020; 1–28. doi: 10.1002/agj2.20433

[22] Buzinaro R, Hugo G, Oliveira F De, Oliveira GHF de, Amaral CB do, Souza Junior CL, et al. Diallel mixed-model analyses to select superior maize parental lines for Azospirillum brasilense and nitrogen-use efficiency. Crop Breed Appl Biotechnol. 2018;18: 382–389. http://dx.doi.org/10.1590

[23] Vivas M, Silveira SF, Viana AP, Amaral AT Jr., Cardoso DL, Pereira MG. Efficiency of circulant diallels via mixed models in the selection of papaya genotypes resistant to foliar fungal diseases. Genet Mol Res. 2014;13: 4797–4804. doi: 10.4238/2014.July.2.9 25062415

[24] Alves RS, de Carvalho Rocha JR do AS, Teodoro PE, de Resende MDV, Henriques EP, Silva LA, et al. Multiple-trait BLUP: a suitable strategy for genetic selection of Eucalyptus. Tree Genet Genomes. 2018;14: 77. doi: 10.1007/s11295-018-1292-7

[25] Carvalho IR, de Pelegrin AJ, Szareski VJ, Ferrari M, da Rosa TC, Martins TS, et al. Diallel and prediction (REML/BLUP) for yield components in intervarietal maize hybrids. Genet Mol Res. 2017;16: 1–12. doi: 10.4238/gmr16039734 28873210

[26] de Souza NO, Alves RS, Teodoro PE, Silva LA, Tardin FD, Tardin AB, et al. Single-and multiple-trait blup in genetic selection of parents and hybrids of grain sorghum. Rev la Fac Ciencias Agrar. 2019;51: 1–12.

[27] Peixouto LS, Nunes JAR, Furtado DF. Factor analysis applied to the G + GE matrix via REML / BLUP for. Crop Breed Appl Biotechnol. 2016;16: 1–6. doi: 10.1590/1984-70332016v16n1a1

[28] Ogut F, Maltecca C, Whetten R, McKeand S, Isik F. Genetic Analysis of Diallel Progeny Test Data Using Factor Analytic Linear Mixed Models. For Sci. 2014;60: 119–127. doi: 10.5849/forsci.12-108

[29] So Y-S, Edwards J. Predictive Ability Assessment of Linear Mixed Models in Multienvironment Trials in Corn. Crop Sci. 2011;51: 542–552. doi: 10.2135/cropsci2010.06.0338

[30] Rocha JR do AS de C, Nunes KV, Carneiro ALN, Marçal TDS, Salvador FV, Carneiro PCS, et al. Selection of superior inbred progenies toward the common bean ideotype. Agron J. 2019;111: 1181–1189. doi: 10.2134/agronj2018.12.0761

[31] Smith A, Cullis B, Thompson R. Analyzing Variety by Environment Data Using Multiplicative Mixed Models and Adjustments for Spatial Field Trend. Biometrics. 2001;57: 1138–1147. doi: 10.1111/j.0006-341x.2001.01138.x 11764254

[32] Burgueño J, de los Campos G, Weigel K, Crossa J. Genomic Prediction of Breeding Values when Modeling Genotype × Environment Interaction using Pedigree and Dense Molecular Markers. Crop Sci. 2012;52: 707–719. doi: 10.2135/cropsci2011.06.0299

[33] Piepho H. Analyzing Genotype-Environment Data by Mixed Models with Multiplicative. Int Biometric Soc. 1997;53: 761–766.

[34] Piepho HP. Ridge Regression and Extensions for Genomewide Selection in Maize. Crop Sci. 2009;49: 1165–1176. doi: 10.2135/cropsci2008.10.0595

[35] Crossa J, Yang R-C, Cornelius PL. Studying crossover genotype × environment interaction using linear-bilinear models and mixed models. J Agric Biol Environ Stat. 2004;9: 362–380. doi: 10.1198/108571104X4423

[36] Crossa J, Burgueño J, Cornelius PL, McLaren G, Trethowan R, Krishnamachari A. Modeling Genotype × Environment Interaction Using Additive Genetic Covariances of Relatives for Predicting Breeding Values of Wheat Genotypes. Crop Sci. 2006;46: 1722–1733. doi: 10.2135/cropsci2005.11–0427

[37] Fritsche-Neto R, Gonçalves MC, Vencovsky R, Souza Junior CL. Prediction of genotypic values of maize hybrids in unbalanced experiments. Crop Breed Appl Biotechnol. 2010;10: 32–39. doi: 10.12702/1984-7033.v10n01a05

[38] Selle ML, Steinsland I, Hickey JM, Gorjanc G. Flexible modelling of spatial variation in agricultural field trials with the R package INLA. Theor Appl Genet. 2019;132: 3277–3293. doi: 10.1007/s00122-019-03424-y 31535162 PMC6820601

[39] Burgueño J, Glaz B, Yeater KM. Chapter 12: Spatial Analysis of Field Experiments. Applied statistics in agricultural, biological, and environmental sciences. 2018. pp. 319–344. doi: 10.2134/appliedstatistics.2016.0011

[40] Cullis BR, Gleeson AC. Spatial Analysis of Field Experiments—An Extension Spatial Analysis to Two Dimensions. Biometrics. 1991;47: 1449–1460.

[41] Gilmour AR, Cullis BR, Verbyla AP. Accounting for Natural and Extraneous Variation in the Analysis of Field Experiments. J Agric Biol Environ Stat. 1997;2: 269–293.

[42] Smith A, Cullis B, Gilmour A. The analysis of crop variety evaluation data in Australia. Aust New Zeal J Stat. 2001;43: 129–145. doi: 10.1111/1467-842X.00163

[43] Gogel B, Smith A, Cullis B. Comparison of a one- and two-stage mixed model analysis of Australia’s National Variety Trial Southern Region wheat data. Euphytica. 2018;214: 44. doi: 10.1007/s10681-018-2116-4

[44] Bernardeli A, Rocha JRAS de C, Borem A, Lorenzoni R, Aguiar R, Silva JNB, et al. Modeling spatial trends and enhancing genetic selection: An approach to soybean seed composition breeding. Crop Sci. 2020; csc2.20364. doi: 10.1002/csc2.20364

[45] Velazco JG, Rodríguez-Álvarez MX, Boer MP, Jordan DR, Eilers PHC, Malosetti M, et al. Modelling spatial trends in sorghum breeding field trials using a two-dimensional P-spline mixed model. Theor Appl Genet. 2017;130: 1375–1392. doi: 10.1007/s00122-017-2894-4 28374049 PMC5487705

[46] Resende MDV de, Duarte JB. Precisão e controle de qualidade em experimentos de avaliação de cultivares. Pesqui Agropecuária Trop. 2007;37: 182–194. doi: 10.5216/pat.v37i3.1867

[47] Lima RO de, Borém A. Melhoramento de Milho. Viçosa—MG: Editora UFV; 2018.

[48] Oliboni R, Faria MV, Neumann M, Resende JTV, Battistelli GM, Tegoni RG, et al. Análise dialélica na avaliação do potencial de híbridos de milho para a geração de populações- base para obtenção de linhagens. Semin Agrar. 2013;34: 7–18. doi: 10.5433/1679-0359.2013v34n1p7

[49] Meirelles WF, Parentoni SN, Guimarães LJM, de Oliveira Guimarães PE, Pacheco CAP, de Oliveira AC, et al. Análise dialélica de linhagens de milho quanto à responsividade ao fósforo e à sua eficiência de uso. Pesqui Agropecu Bras. 2016;51: 224–232. doi: 10.1590/S0100-204X2016000300004

[50] Karim ANMS, Ahmed S, Akhi AH, Talukder MZA, Mujahidi TA. Combining Ability and Heterosis study in maize (Zea mays L.) Hybrids at different environments in Bangladesh. Bangladesh J Agril Res. 2018;7122: 125–134.

[51] Long JK, Bänziger M, Smith ME. Diallel analysis of grain iron and zinc density in southern African-adapted maize inbreds. Crop Sci. 2004;44: 2019–2026. doi: 10.2135/cropsci2004.2019

[52] Sussy M, Ola H, Maria FAB, Niklas BO, Cecilia OM, Willis OK, et al. Micro-spatial analysis of maize yield gap variability and production factors on smallholder farms. Agric. 2019;9: 1–23. doi: 10.3390/agriculture9100219

[53] Qiao CG, Basford KE, DeLacy IH, Cooper M. Evaluation of experimental designs and spatial analyses in wheat breeding trials. Theor Appl Genet. 2000;100: 9–16. doi: 10.1007/s001220050002

[54] Bian L, Zheng R, Su S, Lin H, Xiao H, Wu HX, et al. Spatial analysis increases efficiency of progeny testing of Chinese fir. J For Res. 2017;28: 445–452. doi: 10.1007/s11676-016-0341-z

[55] Fukatsu E, Hiraoka Y, Kuramoto N, Yamada H, Takahashi M. Effectiveness of spatial analysis in Cryptomeria japonica D. Don (sugi) forward selection revealed by validation using progeny and clonal tests. Ann For Sci. 2018;75. doi: 10.1007/s13595-018-0771-1

[56] Chen Z, Helmersson A, Westin J, Karlsson B, Wu HX. Efficiency of using spatial analysis for Norway spruce progeny tests in Sweden. Ann For Sci. 2018;75: 13. doi: 10.1007/s13595-017-0680-8

[57] Liu SM, Constable GA, Cullis BR, Stiller WN, Reid PE. Benefit of spatial analysis for furrow irrigated cotton breeding trials. Euphytica. 2015;201: 253–264. doi: 10.1007/s10681-014-1205-2

[58] DigitalGlobe. Google Earth. http://www.earth.google.com [August 01, 2021]; 2021.

[59] Alvares CA, Stape JL, Sentelhas PC, de Moraes Gonçalves JL, Sparovek G. Köppen’s climate classification map for Brazil. Meteorol Zeitschrift. 2013;22: 711–728. doi: 10.1127/0941-2948/2013/0507

[60] Cruz JC, Karam D, Monteiro MAR, Magalhães PC. A cultura do milho. Sete Lagoas, MG: Embrapa Milho e Sorgo; 2008.

[61] Gilmour AR, Gogel BJ, Cullis BR, Welham Sj, Thompson R. ASReml user guide release 4.1 structural specification. Hemel hempstead VSN Int ltd. 2015.

[62] Verbyla AP. A note on model selection using information criteria for general linear models estimated using REML. Aust New Zeal J Stat. 2019;61: 39–50. doi: 10.1111/anzs.12254

[63] Wilks SS. The Large-Sample Distribution of the Likelihood Ratio for Testing Composite Hypotheses. Ann Math Stat. 1938;9: 60–62. Available: https://www.jstor.org/stable/pdf/2957648.pdf

[64] Griffing B. Concept of General and Specific Combining Ability in relation to diallel crossing systems. Aust J Biol Sci. 1956;9: 463–493. Available: https://www.publish.csiro.au/bi/pdf/BI9560463

[65] Pádua JMV, Das Graças Dias KO, Pastina MM, de Souza JC, Queiroz VAV, da Costa RV, et al. A multi-environment trials diallel analysis provides insights on the inheritance of fumonisin contamination resistance in tropical maize. Euphytica. 2016;211: 277–285. doi: 10.1007/s10681-016-1722-2

[66] Hallauer AR, Carena JM, Miranda Filho JB de. Quantitative genetics in maize breeding. New York: Springer, 2010. 500 p. New York: Springer; 2010.

[67] Resende MDV. Software Selegen-REML/BLUP: a useful tool for plant breeding. Crop Breed Appl Biotechnol. 2016;16: 330–339. doi: 10.1590/1984

[68] Cohen J. A coefficient of agreement for nominal scales. Educ Psychol Meas. 1960;20: 37–46.

[69] Butler DG, Cullis BR, Gilmour AR, Gogel BJ. ASReml-R reference manual (version 3). State Queensland, Dep Prim Ind Fish Brisbane, Qld. 2018.

[70] R Development Core Team. R: A language and environment for statistical computing. Vienna, Austria: R Foundation for Statistical Computing; 2020.

[71] Lado B, Matus I, Rodríguez A, Inostroza L, Poland J, Belzile F, et al. Increased Genomic Prediction Accuracy in Wheat Breeding Through Spatial Adjustment of Field Trial Data. G3 Genes|Genomes|Genetics. 2013;3: 2105–2114. doi: 10.1534/g3.113.007807 24082033 PMC3852373

[72] Rue H, Martino S, Chopin N. Approximate Bayesian inference for latent Gaussian models by using integrated nested Laplace approximations. J R Stat Soc Ser B (Statistical Methodol. 2009;71: 319–392. doi: 10.1111/j.1467-9868.2008.00700.x

[73] Oakey H, Verbyla A, Pitchford W, Cullis B, Kuchel H. Joint modeling of additive and non-additive genetic line effects in single field trials. Theor Appl Genet. 2006;113: 809–819. doi: 10.1007/s00122-006-0333-z 16896718

[74] Bernal-Vasquez AM, Möhring J, Schmidt M, Schönleben M, Schön CC, Piepho HP. The importance of phenotypic data analysis for genomic prediction—a case study comparing different spatial models in rye. BMC Genomics. 2014;15: 1–17. doi: 10.1186/1471-2164-15-1 25087599 PMC4133075

[75] Cooper M, Messina CD, Podlich D, Totir LR, Baumgarten A, Hausmann NJ, et al. Predicting the future of plant breeding: Complementing empirical evaluation with genetic prediction. Crop Pasture Sci. 2014;65: 311–336. doi: 10.1071/CP14007

[76] Borges da Silva ÉD, Xavier A, Faria MV. Joint Modeling of Genetics and Field Variation in Plant Breeding Trials Using Relationship and Different Spatial Methods: A Simulation Study of Accuracy and Bias. Agronomy. 2021;11: 1397. doi: 10.3390/agronomy11071397

[77] Hunt CH, Smith AB, Jordan DR, Cullis BR. Predicting Additive and Non-additive Genetic Effects from Trials Where Traits Are Affected by Interplot Competition. J Agric Biol Environ Stat. 2013;18: 53–63. doi: 10.1007/s13253-012-0117-7

[78] Stroup WW, Baenziger PS, Mulitze DK. Removing Spatial Variation from Wheat Yield Trials: A Comparison of Methods. Crop Sci. 1994;34: 62–66. doi: 10.2135/cropsci1994.0011183X003400010011x

[79] Ramalho MAP, Ferreira DF, Oliveira AC. Experimentação em genética e melhoramento de plantas. 3rd ed. Lavras: Editora UFLA; 2005.

[80] Andrade MHML, Fernandes Filho CC, Fernandes MO, Bastos AJR, Guedes ML, Marçal T de S, et al. Accounting for spatial trends to increase the selection efficiency in potato breeding. Crop Sci. 2020;60: 2354–2372. doi: 10.1002/csc2.20226

[81] Cobb JN, Juma RU, Biswas PS, Arbelaez JD, Rutkoski J, Atlin G, et al. Enhancing the rate of genetic gain in public-sector plant breeding programs: lessons from the breeder’s equation. Theor Appl Genet. 2019;132: 627–645. doi: 10.1007/s00122-019-03317-0 30824972 PMC6439161

[82] Arnhold E, Mora F, Silva RG, Good-god PI V, Rodovalho MA. Evaluation of top-cross popcorn hybrids using mixed linear model methodology. Chil J Agric Res. 2009;69: 46–53. doi: 10.4067/ S0718-58392012000100026

