# Supplementary material for: Accounting for spatial trends in multi-environment diallel analysis in maize breeding
Source: PLoS One. 2021 Oct 21;16(10):e0258473. doi: 10.1371/journal.pone.0258473 (PMC8530354; doi:10.1371/journal.pone.0258473)
Supplement: S1 File — (DOCX) [file pone.0258473.s001.docx]

**Article submission to:** Plos One

**Accounting for Spatial Trends in Multi-Environment Diallel Analysis in Maize Breeding**

Igor Ferreira Coelho^1^ | Marco Antônio Peixoto^1^ | Tiago de Souza Marçal^2^ | Arthur Bernardeli^3^ | Rodrigo Silva Alves^1,4^ | Rodrigo Oliveira de Lima^3^ | Edésio Fialho dos Reis^5^ | Leonardo Lopes Bhering^1*^

^1^ Universidade Federal de Viçosa (UFV), Departamento de Biologia Geral, Viçosa, CEP: 36570-900, Minas Gerais, Brazil

^2^ Universidade Federal de Lavras (UFLA), Departamento de Biologia, Lavras, CEP: 37200-900, Minas Gerais, Brazil

^3^ Universidade Federal de Viçosa (UFV), Departamento de Agronomia, Viçosa, CEP: 36570-900, Minas Gerais, Brazil

^4^ Universidade Federal de Lavras (UFLA), Instituto Nacional de Ciência e Tecnologia do Café (INCT Café), Lavras, CEP: 37200-900, Minas Gerais, Brazil

^5^ Universidade Federal de Jataí (UFJ), Departamento de Agronomia, Jataí, CEP: 75801-615, Goiás, Brazil

^*^ Corresponding author: leonardo.bhering@ufv.br – +55 31 3612-5003

**Table S1. Diallel scheme involving twelve F_2_ hybrids and one rustic genetic material (used as parents) and their respectively crosses (H).**

|  | **1¹** | **2** | **3** | **4** | **5** | **6** | **7** | **8** | **9** | **10** | **11** | **12** | **13** |
| --- | --- | --- | --- | --- | --- | --- | --- | --- | --- | --- | --- | --- | --- |
| **1¹** |  | H1,2 | H1,3 | H1,4 | H1,5 | H1,6 | H1,7 | H1,8 | H1,9 | H1,10 | H1,11 | H1,12 | H1,13 |
| **2** |  |  | H2,3 | H2,4 | H2,5 | H2,6 | H2,7 | H2,8 | H2,9 | H2,10 | H2,11 | H2,12 | H2,13 |
| **3** |  |  |  | H3,4 | H3,5 | H3,6 | H3,7 | H3,8 | H3,9 | H3,10 | H3,11 | H3,12 | H3,13 |
| **4** |  |  |  |  | H4,5 | H4,6 | H4,7 | H4,8 | H4,9 | H4,10 | H4,11 | H4,12 | H4,13 |
| **5** |  |  |  |  |  | H5,6 | H5,7 | H5,8 | H5,9 | H5,10 | H5,11 | H5,12 | H5,13 |
| **6** |  |  |  |  |  |  | H6,7 | H6,8 | H6,9 | H6,10 | H6,11 | H6,12 | H6,13 |
| **7** |  |  |  |  |  |  |  | H7,8 | H7,9 | H7,10 | H7,11 | H7,12 | H7,13 |
| **8** |  |  |  |  |  |  |  |  | H8,9 | H8,10 | H8,11 | H8,12 | H8,13 |
| **9** |  |  |  |  |  |  |  |  |  | H9,10 | H9,11 | H9,12 | H9,13 |
| **10** |  |  |  |  |  |  |  |  |  |  | H10,11 | H10,12 | H10,13 |
| **11** |  |  |  |  |  |  |  |  |  |  |  | H11,12 | H11,13 |
| **12** |  |  |  |  |  |  |  |  |  |  |  |  | H12,13 |
| **13** |  |  |  |  |  |  |  |  |  |  |  |  |  |
|  |  |  |  |  |  |  |  |  |  |  |  |  |  |

¹: Parents 1: P4285; 2: 30F53; 3: P3646; 4: 30K75; 5: RB9110; 6: RB9210; 7: CD384; 8: AS1633; 9: AS1598; 10: 2B587; 11: BM709; 12: DKB390; 13: UFG Rustic Genetic Material.

**Table S2. Hybrids’ information, about type, cycle and kernel texture.**

| Parent | Hybrid / genetic material | Type of Hybrid | Cycle | Kernel Texture |
| --- | --- | --- | --- | --- |
| 1 | P4285^†^ | Simple | Early | Flint |
| 2 | 30F53^†^ | Simple | Early | Semi-Flint |
| 3 | P3646^†^ | Simple | Early | Semi- Flint |
| 4 | 30K75^†^ | Modified simple | Early | Semi- Flint |
| 5 | RB9110 | Simple | Very early | Semi-Dent |
| 6 | RB9210 | Simple | Very early | Flint |
| 7 | CD384 | Triple | Early | Semi- Flint |
| 8 | AS1633^†^ | Simple | Early | Semi- Flint |
| 9 | AS1598 | Simple | Early | Semi- Flint |
| 10 | 2B587 | Simple | Early | Semi-Dent |
| 11 | BM709^†^ | Simple | Semi-early | Semi- Flint |
| 12 | DKB390 | Simple | Early | Semi- Flint |
| 13 | Rustic genetic material^††^ | Synthetic | - | - |

^†^: also adopted as check; ^††^ Rustic genetic material (a synthetic genetic material not yet characterized).

**Table S3. Geographic coordinates, altitude, planting and harvest dates, and temperatures and precipitation information of each trial^†^.**

| Trial | Coordinate | Altitude | Planting date | Harvest date | From planting to harvesting | Average Temperature (°C) ^††^ | |  | Average Precipitation (mm) ^††^ | |  | Sum of Precipitation (mm) ^††^ | |
| --- | --- | --- | --- | --- | --- | --- | --- | --- | --- | --- | --- | --- | --- |
|  |  |  |  |  |  | 0-60 days | All days |  | 0-60 days | All days |  | 0-60 days | All days |
| E1 | 17°55'27.63" S | 685 m | 2/14/2018 | 7/9/2018 | 146 days | 23.31 | 21.66 |  | 4.79 | 2.23 |  | 292.10 | 325.17 |
|  | 51°42'45.51" O |  |  |  |  |  |  |  |  |  |  |  |  |
| E2 | 17°50'4.70" S | 865 m | 2/19/2018 | 7/14/2018 | 146 days | 23.05 | 21.49 |  | 4.57 | 2.06 |  | 279.02 | 300.36 |
|  | 51°29'53.29" O |  |  |  |  |  |  |  |  |  |  |  |  |
| E3 | 17°30'25.06" S | 818 m | 2/24/2018 | 7/11/2018 | 138 days | 22.84 | 21.62 |  | 3.53 | 1.69 |  | 215.49 | 233.86 |
|  | 51°54'35.68" O |  |  |  |  |  |  |  |  |  |  |  |  |
| E4 | 17°23'4.72" S | 938 m | 2/28/2018 | 7/13/2018 | 136 days | 23.01 | 21.79 |  | 3.41 | 1.66 |  | 208.19 | 225.34 |
|  | 52°19'39.01" O |  |  |  |  |  |  |  |  |  |  |  |  |

^†^: These data were obtained from the NASA Langley Research Center (LaRC) Project funded through the NASA Earth Science/Applied Science Program.

^††^: the data of temperature and precipitation are presenting in two sets, from day 0 to day 60^th^ to represent the growing and reproductive cycles, which are the most important in yield, and “All days” to demonstrate the environment information.


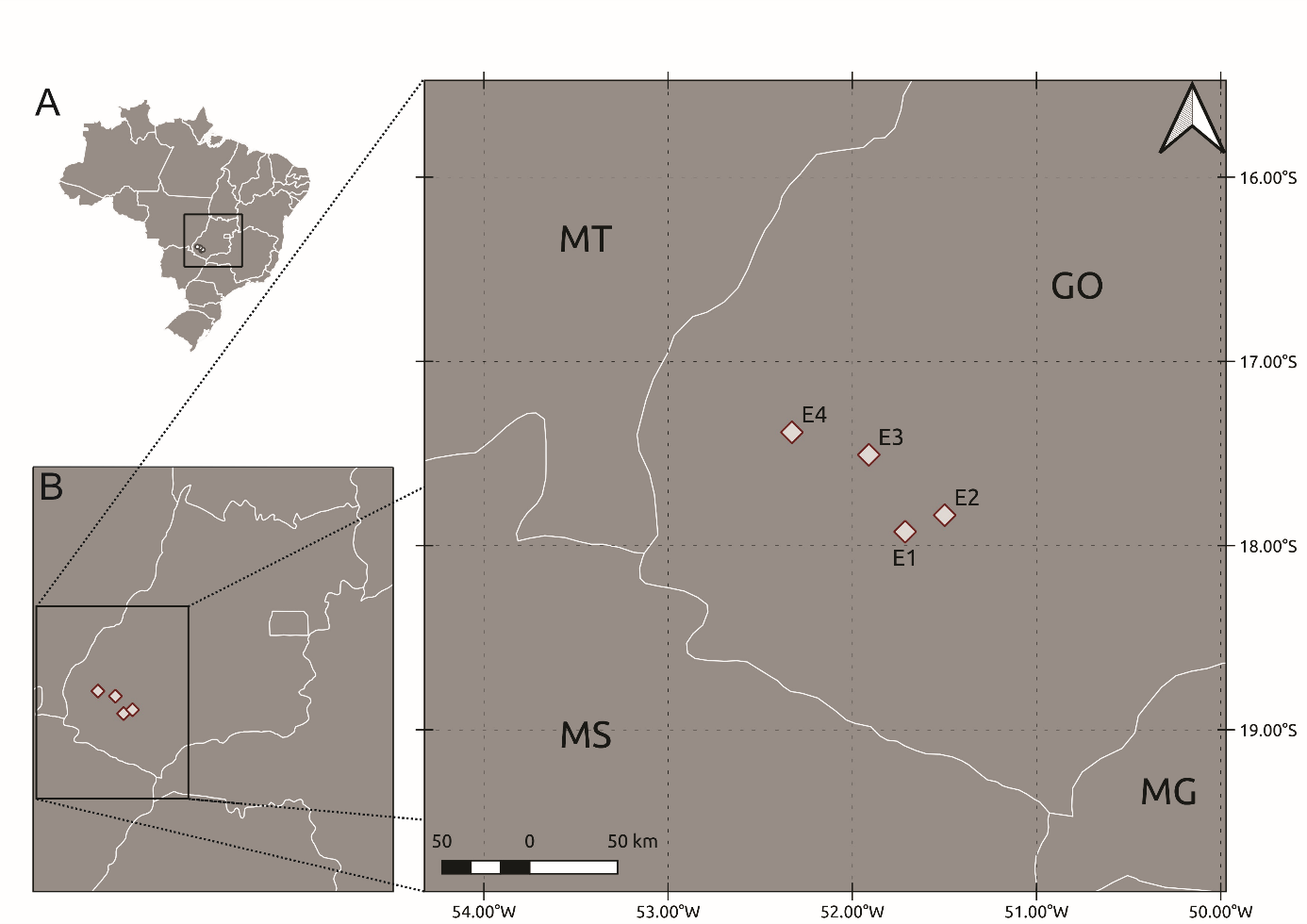


**Figure S1. Map of Brazil (A) detaching the Goiás State (B). The right-side map presents the coordinates, and the localization of all four environments****.** GO = Goiás state, MT = Mato Grosso state, MS = Mato Grosso do Sul state, MG = Minas Gerais state. E1: trial 1, E2: trial 2, E3: trial 3, E4: trial 4

**Table S4. Predicted additive genetic effects in the four trials, by non-spatial (NSPA) and spatial (SPA) analyses.** E1: trial 1, E2: trial 2, E3: trial 3, E4: trial 4.

| Parents^†^ | E1 | | E2 | | E3 | | E4 | |
| --- | --- | --- | --- | --- | --- | --- | --- | --- |
|  | NSPA | SPA | NSPA | SPA | NSPA | SPA | NSPA | SPA |
| P1 | 542.34² | 542.34² | 627.40¹ | 627.40¹ | 70.36 | 75.01 | -2.70 | -0.68 |
| P2 | 102.48 | 102.48 | 202.21 | 202.21 | 24.46 | 98.85 | 82.40 | 66.40 |
| P3 | -389.45 | -389.45 | -164.08 | -164.08 | 114.96³ | 38.09 | 47.69 | 89.50² |
| P4 | -124.34 | -124.34 | -287.39 | -287.39 | -6.71 | 16.14 | 98.79³ | 64.32 |
| P5 | -367.46 | -367.46 | -282.55 | -282.55 | -54.69 | -47.87 | 19.43 | 60.82 |
| P6 | -469.24 | -469.24 | 132.66 | 132.66 | 145.24² | 221.49¹ | 99.41² | 86.03³ |
| P7 | 116.77 | 116.77 | -109.78 | -109.78 | 27.18 | 74.68 | -207.56 | -169.25 |
| P8 | 37.17 | 37.17 | 219.71 | 219.71 | -3.35 | 35.56 | -100.82 | -113.42 |
| P9 | -188.59 | -188.59 | -227.20 | -227.20 | -107.23 | -148.21 | 9.05 | 29.91 |
| P10 | 578.05¹ | 578.05¹ | 245.13² | 245.13² | 145.78¹ | 142.40² | 21.08 | 3.11 |
| P11 | 113.66 | 113.66 | -123.86 | -123.86 | -202.88 | -286.78 | 42.61 | -19.53 |
| P12 | 280.71³ | 280.71³ | 227.56³ | 227.56³ | 68.56 | 130.63³ | 188.68¹ | 191.30¹ |
| P13 | -232.08 | -232.08 | -459.80 | -459.80 | -221.68 | -349.98 | -298.04 | -288.51 |
| Pearson^††^ | 1.00 | | 1.00 | | 0.95 | | 0.97 | |

^†^: Parental F_2_ populations; ^††^: Pearson Correlation Coefficient between NSPA and SPA analyses of each trial; ¹: Highest additive genetic value; ²: Second highest additive genetic value; ³: Third highest additive genetic value.

**Table S5. Hybrids ranking, given by the sum of the predicted additive and dominance genetic effect, in each trial, by non-spatial (NSPA) and spatial (SPA) analyses.** E1 = trial 1, E2 = trial 2, E3 = trial 3, E4 = trial 4.

| Ranking | E1 | | E2 | | E3 | | E4 | |
| --- | --- | --- | --- | --- | --- | --- | --- | --- |
|  | NSPA | SPA | NSPA | SPA | NSPA | SPA | NSPA | SPA |
| 1 | H2,10 | H2,10 | H1,8 | H1,8 | H3,6 | H3,6 | H9,11 | H9,11 |
| 2 | H10,12 | H10,12 | H2,10 | H2,10 | H4,6 | H7,12 | H3,6 | H6,7 |
| 3 | H1,8 | H1,8 | H1,2 | H1,2 | H3,12 | H4,6 | H8,12 | H3,12 |
| 4 | H1,12 | H1,12 | H1,12 | H1,12 | H7,12 | H8,10 | H6,7 | H3,6 |
| 5 | H1,4 | H1,4 | H8,10 | H8,10 | H8,10 | H7,8 | H4,9 | H5,8 |
| 6 | H5,8 | H5,8 | H1,11 | H1,11 | H7,8 | H2,6 | H10,11 | H4,9 |
| 7 | H1,7 | H1,7 | H1,10 | H1,10 | H4,10 | H4,10 | H2,4 | H2,6 |
| 8 | H7,11 | H7,11 | H1,9 | H1,9 | H1,2 | H1,12 | H2,6 | H2,3 |
| 9 | H1,10 | H1,10 | H1,7 | H1,7 | H1,12 | H2,12 | H3,12 | H5,6 |
| 10 | H1,11 | H1,11 | H6,10 | H6,10 | H7,10 | H2,7 | H5,8 | H3,4 |
| 11 | H1,2 | H1,2 | H6,12 | H6,12 | H10,13 | H3,12 | H1,12 | H5,12 |
| 12 | H7,10 | H7,10 | H3,8 | H3,8 | H1,9 | H1,2 | H3,4 | H8,12 |
| 13 | H10,11 | H10,11 | H9,12 | H9,12 | H6,12 | H2,3 | H5,12 | H1,9 |
| 14 | H2,12 | H2,12 | H1,3 | H1,3 | H10,12 | H6,12 | H2,12 | H4,5 |
| 15 | H4,13 | H4,13 | H1,4 | H1,4 | H2,11 | H1,9 | H11,12 | H9,10 |
| 16 | H8,10 | H8,10 | H6,11 | H6,11 | H3,10 | H2,10 | H2,9 | H7,12 |
| 17 | H4,10 | H4,10 | H8,12 | H8,12 | H2,7 | H6,7 | H1,3 | H2,4 |
| 18 | H1,3 | H1,3 | H1,6 | H1,6 | H5,6 | H5,6 | H2,3 | H2,9 |
| 19 | H6,12 | H6,12 | H7,12 | H7,12 | H1,3 | H8,12 | H4,5 | H1,12 |
| 20 | H2,11 | H2,11 | H2,12 | H2,12 | H5,8 | H2,11 | H7,12 | H1,3 |
| 21 | H2,6 | H2,6 | H3,10 | H3,10 | H3,9 | H1,6 | H1,4 | H1,5 |
| 22 | H7,8 | H7,8 | H7,10 | H7,10 | H2,12 | H6,10 | H1,9 | H4,6 |
| 23 | H3,11 | H3,11 | H2,11 | H2,11 | H2,3 | H5,8 | H6,10 | H11,12 |
| 24 | H7,12 | H7,12 | H3,12 | H3,12 | H5,7 | H5,10 | H8,10 | H6,11 |
| 25 | H2,13 | H2,13 | H2,5 | H2,5 | H5,10 | H5,12 | H9,12 | H2,7 |
| 26 | H10,13 | H10,13 | H5,8 | H5,8 | H1,10 | H1,8 | H4,6 | H1,8 |
| 27 | H9,10 | H9,10 | H8,11 | H8,11 | H1,7 | H2,4 | H5,6 | H6,10 |
| 28 | H3,8 | H3,8 | H3,6 | H3,6 | H2,6 | H10,13 | H6,11 | H3,7 |
| 29 | H11,12 | H11,12 | H2,6 | H2,6 | H1,6 | H4,8 | H1,5 | H8,10 |
| 30 | H9,12 | H9,12 | H6,7 | H6,7 | H6,8 | H9,10 | H1,8 | H12,13 |
| 31 | H2,9 | H2,9 | H5,6 | H5,6 | H6,7 | H1,5 | H6,12 | H2,12 |
| 32 | H9,11 | H9,11 | H5,12 | H5,12 | H3,4 | H5,9 | H2,5 | H2,5 |
| 33 | H4,7 | H4,7 | H10,11 | H10,11 | H4,8 | H7,10 | H4,10 | H3,11 |
| 34 | H3,12 | H3,12 | H1,5 | H1,5 | H6,10 | H1,7 | H3,11 | H9,12 |
| 35 | H1,5 | H1,5 | H4,5 | H4,5 | H1,4 | H6,8 | H9,10 | H5,10 |
| 36 | H8,13 | H8,13 | H2,3 | H2,3 | H9,11 | H4,9 | H10,13 | H5,11 |
| 37 | H3,7 | H3,7 | H4,8 | H4,8 | H2,10 | H3,11 | H5,10 | H10,11 |
| 38 | H3,10 | H3,10 | H2,7 | H2,7 | H6,13 | H9,11 | H2,7 | H4,10 |
| 39 | H5,7 | H5,7 | H9,10 | H9,10 | H5,9 | H10,12 | H3,7 | H1,4 |
| 40 | H7,9 | H7,9 | H11,12 | H11,12 | H4,7 | H3,8 | H3,9 | H10,12 |
| 41 | H8,11 | H8,11 | H10,12 | H10,12 | H9,10 | H3,7 | H2,11 | H2,13 |
| 42 | H8,12 | H8,12 | H7,8 | H7,8 | H3,11 | H2,5 | H5,11 | H4,12 |
| 43 | H1,6 | H1,6 | H10,13 | H10,13 | H2,4 | H9,12 | H4,13 | H6,12 |
| 44 | H5,10 | H5,10 | H1,13 | H1,13 | H4,9 | H1,3 | H2,10 | H1,6 |
| 45 | H6,10 | H6,10 | H2,9 | H2,9 | H8,12 | H1,10 | H1,6 | H10,13 |
| 46 | H4,9 | H4,9 | H3,4 | H3,4 | H3,7 | H3,9 | H10,12 | H2,10 |
| 47 | H11,13 | H11,13 | H4,6 | H4,6 | H5,11 | H1,4 | H3,8 | H3,9 |
| 48 | H4,5 | H4,5 | H8,13 | H8,13 | H5,12 | H4,7 | H2,13 | H3,8 |
| 49 | H5,12 | H5,12 | H7,9 | H7,9 | H3,13 | H4,5 | H4,12 | H8,11 |
| 50 | H2,3 | H2,3 | H3,11 | H3,11 | H2,9 | H3,10 | H12,13 | H8,9 |
| 51 | H7,13 | H7,13 | H2,4 | H2,4 | H3,8 | H3,4 | H1,11 | H1,11 |
| 52 | H2,4 | H2,4 | H6,8 | H6,8 | H2,5 | H5,11 | H4,8 | H4,7 |
| 53 | H1,9 | H1,9 | H6,13 | H6,13 | H9,12 | H8,13 | H8,11 | H3,10 |
| 54 | H1,13 | H1,13 | H8,9 | H8,9 | H1,5 | H5,7 | H4,11 | H4,13 |
| 55 | H6,13 | H6,13 | H4,7 | H4,7 | H1,8 | H2,9 | H1,2 | H9,13 |
| 56 | H4,8 | H4,8 | H5,13 | H5,13 | H6,11 | H6,11 | H3,10 | H1,10 |
| 57 | H9,13 | H9,13 | H5,10 | H5,10 | H8,11 | H8,9 | H7,11 | H1,2 |
| 58 | H5,9 | H5,9 | H2,8 | H2,8 | H8,13 | H4,12 | H1,10 | H1,7 |
| 59 | H6,11 | H6,11 | H3,7 | H3,7 | H8,9 | H7,9 | H11,13 | H11,13 |
| 60 | H5,11 | H5,11 | H9,11 | H9,11 | H4,12 | H11,13 | H5,7 | H2,11 |
| 61 | H3,4 | H3,4 | H7,11 | H7,11 | H1,11 | H7,13 | H1,7 | H4,8 |
| 62 | H3,9 | H3,9 | H5,11 | H5,11 | H4,5 | H3,13 | H6,9 | H5,7 |
| 63 | H6,8 | H6,8 | H2,13 | H2,13 | H11,13 | H6,13 | H4,7 | H3,5 |
| 64 | H12,13 | H12,13 | H4,12 | H4,12 | H2,13 | H1,13 | H8,9 | H7,10 |
| 65 | H4,12 | H4,12 | H12,13 | H12,13 | H12,13 | H12,13 | H7,10 | H4,11 |
| 66 | H6,9 | H6,9 | H6,9 | H6,9 | H1,13 | H8,11 | H5,9 | H7,11 |
| 67 | H5,13 | H5,13 | H9,13 | H9,13 | H4,13 | H10,11 | H3,5 | H5,9 |
| 68 | H2,5 | H2,5 | H7,13 | H7,13 | H5,13 | H4,13 | H9,13 | H5,13 |
| 69 | H4,6 | H4,6 | H3,9 | H3,9 | H7,9 | H1,11 | H6,8 | H6,8 |
| 70 | H8,9 | H8,9 | H4,10 | H4,10 | H10,11 | H2,13 | H6,13 | H8,13 |
| 71 | H2,7 | H2,7 | H4,13 | H4,13 | H7,13 | H5,13 | H2,8 | H6,13 |
| 72 | H6,7 | H6,7 | H4,9 | H4,9 | H6,9 | H7,11 | H8,13 | H1,13 |
| 73 | H5,6 | H5,6 | H4,11 | H4,11 | H7,11 | H4,11 | H5,13 | H7,9 |
| 74 | H4,11 | H4,11 | H5,9 | H5,9 | H4,11 | H6,9 | H1,13 | H6,9 |
| 75 | H2,8 | H2,8 | H5,7 | H5,7 | H2,8 | H11,12 | H7,9 | H2,8 |
| 76 | H3,13 | H3,13 | H11,13 | H11,13 | H3,5 | H3,5 | H3,13 | H3,13 |
| 77 | H3,6 | H3,6 | H3,13 | H3,13 | H11,12 | H2,8 | H7,8 | H7,8 |
| 78 | H3,5 | H3,5 | H3,5 | H3,5 | H9,13 | H9,13 | H7,13 | H7,13 |

**Table S6. Dominance genetic effect for the four environments, by non-spatial (NSPA) and spatial (SPA) approaches.** E1: trial 1, E2: trial 2, E3: trial 3, E4: trial 4.

| Interpopulational Hybrids^†^ | E1 | | E2 | | E3 | | E4 | |
| --- | --- | --- | --- | --- | --- | --- | --- | --- |
|  | NSPA | SPA | NSPA | SPA | NSPA | SPA | NSPA | SPA |
| H1,2 | 108.91 | 108.91 | 192.95^#^ | 192.95^#^ | 203.32^#^ | 163.28 | -170.30 | -199.75 |
| H1,3 | 208.08 | 208.08 | 0.48 | 0.48 | 60.67 | -60.76 | 157.22 | 152.54 |
| H1,4 | 349.53^#^ | 349.53^#^ | 52.38 | 52.38 | 19.47 | -63.21 | 95.52 | 10.99 |
| H1,5 | 10.11 | 10.11 | -130.41 | -130.41 | -71.50 | 44.81 | 92.82 | 126.43 |
| H1,6 | -74.91 | -74.91 | -194.77 | -194.77 | -15.89 | 44.89 | -92.21 | -54.75 |
| H1,7 | 184.34 | 184.34 | 95.54 | 95.54 | 62.80 | -48.28 | -51.85 | -92.60 |
| H1,8 | 436.05^#^ | 436.05^#^ | 323.31^#^ | 323.31^#^ | -105.72 | 104.44 | 147.56 | 169.70 |
| H1,9 | -358.95 | -358.95 | 165.98 | 165.98 | 226.17^#^ | 270.22^#^ | 131.22 | 231.25^#^ |
| H1,10 | -90.53 | -90.53 | -65.14 | -65.14 | 4.49 | -113.88 | -148.51 | -165.19 |
| H1,11 | 120.71 | 120.71 | 136.00 | 136.00 | -45.40 | -230.94 | -112.57 | -126.07 |
| H1,12 | 239.88 | 239.88 | 168.70 | 168.70 | 160.96 | 239.71^#^ | 142.12 | 123.96 |
| H1,13 | -343.45 | -343.45 | -129.03 | -129.03 | -170.98 | -109.60 | -198.65 | -179.11 |
| H2,3 | 10.58 | 10.58 | 10.80 | 10.80 | 67.37 | 181.47 | 106.31 | 210.65 |
| H2,4 | -166.08 | -166.08 | -48.30 | -48.30 | -17.21 | 94.65 | 186.24^#^ | 157.87 |
| H2,5 | -267.50 | -267.50 | 170.84^#^ | 170.84^#^ | -40.17 | -27.91 | 40.30 | 28.08 |
| H2,6 | 422.97^#^ | 422.97^#^ | -73.69 | -73.69 | 17.91 | 219.01 | 178.82 | 250.61 |
| H2,7 | -568.11 | -568.11 | -30.56 | -30.56 | 148.94 | 176.11 | 68.65 | 164.56 |
| H2,8 | -792.58 | -792.58 | -368.10 | -368.10 | -520.75 | -726.08 | -239.15 | -341.16 |
| H2,9 | 179.11 | 179.11 | -48.68 | -48.68 | -0.94 | -87.95 | 135.14 | 174.46 |
| H2,10 | 607.03^#^ | 607.03^#^ | 437.70^#^ | 437.70^#^ | -43.74 | 96.46 | -87.56 | -74.29 |
| H2,11 | 148.93 | 148.93 | 100.47 | 100.47 | 276.09^#^ | 293.54^#^ | -88.09 | -205.95 |
| H2,12 | 215.58 | 215.58 | -46.02 | -46.02 | 95.37 | 150.89 | 54.31 | -34.88 |
| H2,13 | 250.38 | 250.38 | -98.87 | -98.87 | -72.04 | -216.31 | 48.07 | 124.38 |
| H3,4 | -42.22 | -42.22 | 160.71 | 160.71 | 12.17 | -74.31 | 146.93 | 196.18 |
| H3,5 | -607.57 | -607.57 | -565.07 | -565.07 | -588.10 | -635.32 | -229.08 | -280.13 |
| H3,6 | -508.47 | -508.47 | 110.77 | 110.77 | 567.90^#^ | 624.73^#^ | 255.39 | 309.19 |
| H3,7 | 157.86 | 157.86 | -37.17 | -37.17 | -102.72 | -51.83 | 65.80 | 143.71 |
| H3,8 | 326.56 | 326.56 | 284.31^#^ | 284.31^#^ | -107.64 | -30.84 | -28.30 | -59.42 |
| H3,9 | -11.00 | -11.00 | -132.56 | -132.56 | 140.57 | 45.12 | -50.83 | -110.78 |
| H3,10 | -100.40 | -100.40 | 125.57 | 125.57 | 45.49 | -133.22 | -167.72 | -186.57 |
| H3,11 | 331.92^#^ | 331.92^#^ | 53.48 | 53.48 | 54.54 | 138.95 | 8.05 | 43.72 |
| H3,12 | 163.67 | 163.67 | 103.11 | 103.11 | 372.60^#^ | 167.54 | 132.73 | 268.65 |
| H3,13 | -496.15 | -496.15 | -275.51 | -275.51 | 13.74 | -49.32 | -261.79 | -344.61 |
| H4,5 | 143.90 | 143.90 | 323.05^#^ | 323.05^#^ | -95.30 | -24.43 | 110.06 | 178.87 |
| H4,6 | -107.04 | -107.04 | 10.61 | 10.61 | 406.75^#^ | 483.55^#^ | 23.80 | 72.44 |
| H4,7 | 132.60 | 132.60 | 52.36 | 52.36 | 25.81 | -69.43 | -108.46 | -84.46 |
| H4,8 | -180.01 | -180.01 | 54.63 | 54.63 | 69.88 | 59.47 | -94.35 | -161.88 |
| H4,9 | 61.67 | 61.67 | -124.42 | -124.42 | 41.26 | 81.85 | 236.39 | 292.51 |
| H4,10 | 69.55 | 69.55 | -337.53 | -337.53 | 190.42 | 268.53^#^ | -1.34 | 10.55 |
| H4,11 | -563.52 | -563.52 | -225.80 | -225.80 | -373.95 | -319.61 | -183.61 | -268.69 |
| H4,12 | -443.84 | -443.84 | -210.98 | -210.98 | -138.10 | -231.76 | -205.17 | -121.23 |
| H4,13 | 564.40^#^ | 564.40^#^ | 11.11 | 11.11 | -172.50 | -153.53 | 73.05 | -36.51 |
| H5,6 | -138.72 | -138.72 | 163.67 | 163.67 | 111.40 | 116.78 | 58.49 | 200.89 |
| H5,7 | 100.05 | 100.05 | -353.86 | -353.86 | 149.71 | -117.17 | -49.82 | -138.65 |
| H5,8 | 680.53^#^ | 680.53^#^ | 139.80 | 139.80 | 179.60 | 183.68 | 290.07 | 394.58 |
| H5,9 | 34.02 | 34.02 | -193.97 | -193.97 | 117.75 | 138.64 | -196.29 | -310.59 |
| H5,10 | -146.59 | -146.59 | -135.52 | -135.52 | 83.90 | 127.78 | 6.46 | 28.00 |
| H5,11 | -163.26 | -163.26 | -21.48 | -21.48 | 95.12 | 102.63 | -57.38 | 25.32 |
| H5,12 | -85.49 | -85.49 | 103.52 | 103.52 | -44.62 | 123.45 | 112.97 | 144.72 |
| H5,13 | -94.60 | -94.60 | 222.01^#^ | 222.01^#^ | -153.07 | -186.53 | -123.72 | -164.34 |
| H6,7 | -339.79 | -339.79 | 77.32 | 77.32 | -13.20 | 62.24 | 347.66 | 457.21 |
| H6,8 | -131.83 | -131.83 | -267.71 | -267.71 | 7.07 | -112.61 | -205.33 | -283.38 |
| H6,9 | -65.20 | -65.20 | -207.71 | -207.71 | -438.54 | -541.79 | -214.04 | -419.06 |
| H6,10 | -144.84 | -144.84 | 137.65 | 137.65 | -88.70 | 4.59 | 74.10 | 64.02 |
| H6,11 | -97.70 | -97.70 | 197.74^#^ | 197.74^#^ | -54.02 | -97.61 | 33.35 | 80.07 |
| H6,12 | 357.95^#^ | 357.95^#^ | 132.67 | 132.67 | 100.45 | 71.35 | -48.81 | -145.25 |
| H6,13 | 144.24 | 144.24 | 43.68 | 43.68 | 76.78 | -164.47 | -130.43 | -202.16 |
| H7,8 | 149.57 | 149.57 | -81.15 | -81.15 | 258.56^#^ | 355.85^#^ | -326.61 | -313.50 |
| H7,9 | 9.98 | 9.98 | 78.95 | 78.95 | -254.12 | -137.24 | -252.47 | -285.54 |
| H7,10 | 71.34 | 71.34 | 72.02 | 72.02 | 139.47 | -80.57 | -86.84 | -134.25 |
| H7,11 | 355.73^#^ | 355.73^#^ | -87.80 | -87.80 | -370.82 | -313.41 | -52.39 | -169.60 |
| H7,12 | -5.66 | -5.66 | 114.89 | 114.89 | 393.38^#^ | 521.78^#^ | 155.87 | 212.78 |
| H7,13 | -77.88 | -77.88 | -8.32 | -8.32 | -310.98 | -58.44 | -295.84 | -408.60 |
| H8,9 | -338.73 | -338.73 | -127.48 | -127.48 | -49.89 | -88.85 | -117.90 | -86.67 |
| H8,10 | 46.92 | 46.92 | 188.04^#^ | 188.04^#^ | 301.18^#^ | 347.66^#^ | 169.53 | 158.99 |
| H8,11 | -107.04 | -107.04 | 48.61 | 48.61 | 2.61 | -168.31 | -72.04 | -18.46 |
| H8,12 | -193.96 | -193.96 | -30.98 | -30.98 | -60.52 | 119.43 | 252.13 | 207.58 |
| H8,13 | 158.63 | 158.63 | 52.43 | 52.43 | 9.97 | 70.27 | -60.42 | -101.24 |
| H9,10 | -28.48 | -28.48 | -7.46 | -7.46 | 10.49 | 75.12 | 32.60 | 211.65 |
| H9,11 | 170.90 | 170.90 | -1.64 | -1.64 | 197.52 | 231.04^#^ | 343.53 | 489.00 |
| H9,12 | 93.77 | 93.77 | 307.36^#^ | 307.36^#^ | -42.39 | 5.48 | 30.49 | -47.42 |
| H9,13 | -21.73 | -21.73 | 68.57 | 68.57 | -448.36 | -467.20 | -52.29 | -24.12 |
| H10,11 | 66.10 | 66.10 | -1.74 | -1.74 | -300.14 | -240.06 | 245.00 | 53.53 |
| H10,12 | 496.86^#^ | 496.86^#^ | -236.04 | -236.04 | 91.20 | -126.12 | -155.60 | -66.18 |
| H10,13 | -5.19 | -5.19 | 63.12 | 63.12 | 246.37^#^ | 230.62 | 179.40 | 111.68 |
| H11,12 | -55.32 | -55.32 | -50.82 | -50.82 | -491.60 | -460.28 | 68.85 | 47.32 |
| H11,13 | -41.93 | -41.93 | -268.64 | -268.64 | 63.12 | 143.89 | -12.34 | -25.04 |
| H12,13 | -374.67 | -374.67 | -132.00 | -132.00 | -116.74 | -162.34 | -6.93 | 143.43 |
| Pearson ^††^ | 1.00 | | 1.00 | | 0.88 | | 0.93 | |
| Cohen’s Kappa ^†††^ | 1.00 | | 1.00 | | 0.66 | | 0.54 | |

^†^: Interpopulation hybrids generated by parents’ crossing *i* and *j* (H*i*,*j*); ^††^: Pearson correlation coefficient between NSPA and SPA analyses; ^†††^: Cohen’s Kappa coefficient between NSPA and SPA analyses; ^#^10 highest predicted dominance genetic effects.
